# Hybrid spectral CT reconstruction

**DOI:** 10.1371/journal.pone.0180324

**Published:** 2017-07-06

**Authors:** Darin P. Clark, Cristian T. Badea

**Affiliations:** Center for In Vivo Microscopy, Department of Radiology, Duke University Medical Center, Durham, NC, United States of America; Beijing University of Technology, CHINA

## Abstract

Current photon counting x-ray detector (PCD) technology faces limitations associated with spectral fidelity and photon starvation. One strategy for addressing these limitations is to supplement PCD data with high-resolution, low-noise data acquired with an energy-integrating detector (EID). In this work, we propose an iterative, hybrid reconstruction technique which combines the spectral properties of PCD data with the resolution and signal-to-noise characteristics of EID data. Our hybrid reconstruction technique is based on an algebraic model of data fidelity which substitutes the EID data into the data fidelity term associated with the PCD reconstruction, resulting in a joint reconstruction problem. Within the split Bregman framework, these data fidelity constraints are minimized subject to additional constraints on spectral rank and on joint intensity-gradient sparsity measured between the reconstructions of the EID and PCD data. Following a derivation of the proposed technique, we apply it to the reconstruction of a digital phantom which contains realistic concentrations of iodine, barium, and calcium encountered in small-animal micro-CT. The results of this experiment suggest reliable separation and detection of iodine at concentrations ≥ 5 mg/ml and barium at concentrations ≥ 10 mg/ml in 2-mm features for EID and PCD data reconstructed with inherent spatial resolutions of 176 μm and 254 μm, respectively (point spread function, FWHM). Furthermore, hybrid reconstruction is demonstrated to enhance spatial resolution within material decomposition results and to improve low-contrast detectability by as much as 2.6 times relative to reconstruction with PCD data only. The parameters of the simulation experiment are based on an *in vivo* micro-CT experiment conducted in a mouse model of soft-tissue sarcoma. Material decomposition results produced from this *in vivo* data demonstrate the feasibility of distinguishing two K-edge contrast agents with a spectral separation on the order of the energy resolution of the PCD hardware.

## Introduction

Photon counting x-ray detector technology promises to revolutionize both clinical and preclinical x-ray CT imaging applications; current x-ray CT systems, however, are largely based on energy integrating x-ray detectors (EIDs), which integrate incoming x-rays over a polychromatic source spectrum. EIDs have proven to be a robust and reliable technology for spectral imaging with dual-energy (two kVp) CT. Clinically, dual-energy CT is now established in many diagnostic imaging applications, including the characterization of vascular diseases, lung perfusion and ventilation, and kidney stones [1]. Preclinically, many translational applications of EID-based spectral CT have been developed, including the characterization of myocardial infarction [2], atherosclerotic plaque composition [3], and tumor aggressiveness and therapy response in primary sarcoma tumors [4] and in lung cancer [5]. These preclinical applications typically use one (or more) contrast agent(s) based on a heavy metal (e.g. iodine, barium, gold) which can be semi-quantitatively separated from soft tissues based on its spectral signature [6, 7]. Despite these promising applications, future advancements in spectral CT imaging and quantitative material differentiation are largely limited by the spectral sensitivity of EIDs. Nominally, photon counting x-ray detector (PCD) technology delivers superior spectral sensitivity to EIDs by binning incoming x-ray photons as a function of their energy, achieving detector-centric spectral differentiation with a single, polychromatic source spectrum.

Current PCD hardware uses a semiconductor sensor (e.g. silicon, CdTe, CdZnTe) which absorbs energy from incident x-rays through scattering and absorption interactions. Free electrons within the semiconductor material, which are produced by these interactions, are read out by a pixelated anode. The resulting electrical signal is thresholded based on its amplitude as an indirect measure of the energy of the incident x-ray photon. Unfortunately, the recorded energy of x-ray photons is often distorted due to technical challenges associated with the read-out electronics and to physical phenomena such as charge sharing, pulse pileup, and K-escape [8]. Addressing these challenges and meeting the design goals of specific clinical and preclinical imaging applications has led to a number detector designs. For instance, a silicon-based PCD with 50 μm^2^ pixels and slits to reduce scatter has been integrated into a commercial mammography system (MicroDose mammography; Koninklijke Philips N.V., Amsterdam, Netherlands). More recently, a dual-source clinical CT scanner produced by Siemens (Munich, Germany) and installed at the Mayo Clinic (Rochester, MN) was modified, replacing one of its EIDs with a PCD. The CdTe-based photon counting detector has 0.5-mm detector pixels (effective size, z dimension) and covers a 275-mm field of view. Preliminary cadaver studies performed with this hybrid clinical scanner demonstrated improved signal-to-noise characteristics and reduced beam-hardening and calcium-blooming artifacts relative to clinical EID data at diagnostically relevant tube currents and kVp settings [9].

PCD-based preclinical CT, which we focus on in this work, requires much higher spatial resolution than clinical CT imaging (micro-CT; typical voxel sizes: ~5–100 μm^3^). When high-resolution imaging is performed with a low-power, micro-focus x-ray source and a small detector pixel pitch, common PCD issues associated with count-rate performance and charge collection efficiency can be mitigated. However, PCD-based micro-CT suffers from high noise levels due to photon starvation encountered with small pixels and energy binning. Spectral distortions due to charge sharing between small detector pixels (<<0.5 mm^2^) are also a significant [8]. It follows that PCD-based micro-CT system design requires a balance between design parameters related to these issues to achieve an optimum level of performance within the resolution constraints. Currently, there is one commercially available PCD-based micro-CT system, the Medipix All Resolution System (MARS Bioimaging Ltd.; Christchurch, New Zealand) [10]. The MARS is based on the Medipix3 detector chip developed at CERN (Geneva, Switzerland). In addition to its 55 μm^2^ (110 μm^2^) pixel pitch, a unique feature of the Medipix3 detector is its charge-summing circuitry which can be used to compensate for charge sharing between neighboring detector pixels [11]. Several other custom-built, PCD-based micro-CT scanners have been demonstrated [12–16].

In both the clinical and preclinical arenas, hardware development will continue to improve semiconductor readouts, count-rate performance, field-of-view coverage, etc.; however, fundamental issues such as photon starvation, quantitative material decomposition accuracy, and the cost of PCDs will require joint consideration between hardware development and the algorithms used for post-processing and reconstruction. A prevailing strategy for overcoming many of these issues is to supplement PCD projection data with projection data from a second EID detector. This hybrid data acquisition strategy has already been implemented to extend the field of view of PCD projection data to avoid truncation and to provide context for experimental PCD data [9, 12, 13, 17].

Following from these implementations and with the objective of *in vivo*, preclinical micro-CT imaging, we focus on the problem of jointly reconstructing EID and PCD data when the PCD data is lower-resolution and noisier than the EID data. In other words, we propose and demonstrate a hybrid spectral CT reconstruction technique which combines the spectral contrast of the PCD data with the spatial resolution of the EID data, yielding high-resolution spectral CT reconstructions. While challenging from an algorithmic perspective, this hybrid imaging paradigm is very attractive for *in vivo* micro-CT. By easing the pixel size requirements on the PCD, problems such as spectral distortion due to charge sharing become more manageable, while the signal-to-noise characteristics of the PCD data improve. Economically, this paradigm also makes sense given the potential to co-develop PCD detectors for both clinical and preclinical imaging.

### Resolution enhancement in hybrid CT

In digital image processing literature, the problem of synthesizing high-resolution spectral data from high-resolution “black and white” (panchromatic, “pan”) imagery and lower-resolution “color” (spectrally resolved, “spectral”) imagery is known as pansharpening. Many techniques for pansharpening have been proposed, combined, and successfully applied to 2D digital images, including the following broad categories: (1) color space transformations and component substitution [18, 19]; (2) forward modeling and inversion of the imaging process [20]; and (3) multi-resolution analysis and wavelet fusion [18, 19]. In this work, we adapt concepts from each of these three categories to perform hybrid CT reconstruction.

The objective of component substitution (1) is to enhance low-resolution spectral data with structural details from high-resolution pan data. Separation of structural details from spectral information in both the pan and spectral data is achieved through a color space transformation. In this transformed space, the structural details from the high-resolution pan data are substituted for the structural details of the low-resolution spectral data, yielding structurally enhanced spectral data. Enhanced spectral CT data can be analogously synthesized from high-resolution EID data and low-resolution PCD data through material decomposition. Specifically, material decomposition of PCD data, which can be performed given sufficient spectral contrast, transforms the PCD data into energy-independent material maps. These material maps can then be recomposed to produce a low-resolution estimate of the EID data. Replacing this low-resolution estimate with the actual EID data enhances structural details in the PCD data. In dose-agnostic imaging applications (e.g. *ex vivo* imaging, non-destructive testing), analytical reconstruction combined with material-based component substitution could provide a closed-form solution for the reconstruction of hybrid CT data; however, we deal with a dose-limited *in vivo* application in this work. Details on our implementation of material-based component substitution and the role it plays in our iterative reconstruction technique for hybrid CT are provided in the *Methods* section.

Our iterative, algebraic reconstruction technique is based on a simplistic, but largely intuitive, forward model (2). Specifically, the EID imaging chain consists of a polychromatic x-ray source and an EID. The source-detector geometry is described by a system matrix for each sampled angle. Following sampling and prior to reconstruction, gain correction and log-transformation are applied to the EID projection data. With the addition of detector binning, acquisition of the PCD projection data is similarly performed with the PCD imaging chain; however, the system matrix for the PCD chain is defined relative to the EID chain, including an affine transform which maps the PCD line integrals from the space of the EID imaging chain to the space of the PCD imaging chain. Defining the PCD system matrix relative to the EID imaging chain yields PCD reconstructions in the space of the EID reconstruction, a prerequisite for iterative hybrid reconstruction. Furthermore, the resolution of the PCD and EID reconstructions are defined relative to the resolution of the EID data. A resampling operator reduces the spatial resolution of the PCD reconstruction to match the resolution of the PCD imaging chain prior to projection with the system matrix. The forward model further incorporates component substitution by defining the high-resolution estimate of the PCD data as the sum of the high-resolution EID data and the spectral contrast from the PCD data. Mathematical details of our proposed forward model are provided in the *Methods* section.

Finally, multi-resolution considerations (3) are incorporated into our proposed hybrid reconstruction technique in two ways. The first way, as previously discussed, is the resampling operation applied in the forward model. Inversion of the forward model requires deblurring of the PCD reconstructions to match the spatial resolution of the EID data. This inversion process is very sensitive to noise in the PCD projection data, requiring regularization and leading to the second way in which we incorporate multi-resolution considerations. Specifically, to deal with low-frequency, correlated noise introduced by the upsampling and deblurring of the low-resolution PCD data, we take inspiration from an algorithm closely related to pansharpening: super-resolution. Super-resolution combines several aliased, low-resolution images with different sampling patterns into a single, high-resolution image [21]. As we will show, correlated noise can be robustly addressed through joint regularization of the EID and PCD data following a reversible aliasing operation which improves denoising performance at low spatial frequencies. In the next section, we establish a context for our regularization strategy relative to other, previously proposed strategies for the regularization of spectral CT data.

### Rank and sparsity constraints in spectral CT reconstruction

Given the inherent trade-offs between x-ray dose and photon flux per spectral bin in *in vivo* PCD-based CT, robust regularization is generally required for accurate reconstruction and material decomposition. There are two main categories of regularization which have proven effective in spectral CT problems (dual energy and PCD CT): (1) regularization based on intensity-gradient sparsity constraints and (2) regularization based on structural redundancy (rank) constraints. Intensity-gradient sparsity constraints (1) are either applied directly to numerical gradients computed from the reconstructed intensity values or indirectly to intensity values processed with a kernel or sparsifying transform. Directly enforcing intensity-gradient sparsity implicitly assumes that the underlying image structure is piece-wise constant, since only edge information which is significantly differentiated from noise is preserved. Prominent examples of direct sparsity constraints include total variation minimization [22] and the differentiable Huber roughness penalty [23]. These direct methods benefit from high computational efficiency, but generally demonstrate inferior performance relative to well-optimized, indirect methods which consider higher-order (more distant) intensity differences (e.g. kernel methods) or which sample the data with a multi-resolution, sparsifying transform. Prominent examples of indirect sparsity constraints include bilateral total variation minimization [7], non-local means [24, 25], weighted intensity averaging over large-scale neighborhoods [26], and soft thresholding of redundant, tight-frame transform coefficients [27]. Among sparsity constraints which have applied to spectral CT reconstruction problems, non-local means, and more generally dictionary learning and sparse coding, are somewhat unique because they do not implicitly assume or require piece-wise constant image structure [28].

In many recent spectral CT reconstruction papers, intensity-gradient sparsity constraints are often supplemented with an additional structural redundancy constraint (2). Redundancy constraints exploit the coherence in image structure between spatially localized spectral samples to further improve the robustness of regularization. As with direct sparsity constraints, direct redundancy constraints provide computationally efficient methods for exploiting spectral redundancy and include methods such as energy-weighted averaging [29], prior image constrained compressed sensing (PICCS) [30], and local highly constrained backprojection reconstruction (HYPR-LR) [31]. More computationally expensive, indirect methods operate on spectral singular value decompositions, either treating each reconstructed volume as a whole (e.g. the prior rank, intensity, and sparsity model; PRISM [27]) or by parsing each volume into spatially-matching spectral patches [32]. Both methods have advantages when the number of spectral measurements exceeds the number of unique basis materials. Working with whole volumes provides significant, non-local statistical power in assigning voxels to singular vectors whose contrast is dominated by a subset of basis materials. Conversely, patches offer reduced statistical power in grouping voxels within singular vectors, but can yield meaningful rank reduction even when the number of spectral samples is less than the number of unique basis materials, since the local rank tends to be lower (consists of fewer materials) than the global rank.

An emerging category of spectral regularization strategies combines sparse representation with the expectation of low spectral rank in a single constraint (rank-sparsity constraints). Intuitively, concentrating CT data into a small number of significant, robustly determined spatial coefficients through intensity differentiation (sparsifying transformation) and then penalizing disagreement in those coefficients between energies is a powerful paradigm for regularization. For example, total nuclear variation minimization not only directly enforces piece-wise constant image structure through traditional total variation minimization, it enforces matching edge information between spectral channels by thresholding singular values associated with the total variation gradients computed for each spectral channel [33]. Following from the previous discussion, moving from direct to indirect enforcement of sparsity within the context of rank-sparsity can further improve performance, at the expense of increased computation. For example, a variant of the PRISM algorithm combines a multi-resolution, tight frame transformation with the thresholding of global singular values [27]. Unfortunately, for many real-world, multi-dimensional CT reconstruction problems compounding the algorithmic complexity of rank-sparsity constraints with the computation and storage requirements of an indirect sparsity constraint—e.g. storing 7–8 sets of redundant transform coefficients per resolution level and energy (3D)—severely limits practical application. Furthermore, soft thresholding methods, which are frequently used to enforce sparsity and low rank, can require a significant number of iterations to converge (100+) due to the balance between a static thresholding parameter and the potential for bias in the final result [34].

In this work, we further develop our own previously proposed strategy for regularizing iterative spectral CT reconstruction problems: rank-sparse kernel regression (RSKR) [35, 36]. As detailed in the *Methods* sections, RSKR combines joint bilateral total variation minimization [7], singular value decomposition, and adaptively determined regularization parameters within the split Bregman method [37] to enforce low structural rank and intensity gradient sparsity between spectral channels. The result is highly robust spectral regularization with rapid convergence and without the need to store redundant information (e.g. redundant transform coefficients) between iterations. To deal with correlated noise in the problem of hybrid reconstruction, we integrate an aliasing strategy into RSKR, which improves robustness in removing low frequency noise without significantly increasing computation time or algorithm complexity. In tandem with RSKR, we also perform regularization based on dictionary learning and sparse coding. As mentioned earlier, dictionary learning methods adapt to the inherent signal model represented in the patches used for training. In the hybrid reconstruction problem, training a redundant dictionary on the high-resolution EID data explicitly models the spatial resolution characteristics of the EID data, which can then be used to code the hybrid data in a way that is consistent with the EID data. For both RSKR and dictionary sparse coding, computational speed and scalability to practical reconstruction problems are achieved through GPU-based implementations of RSKR and dictionary sparse coding.

### Contributions

Our aim in this work is to make several meaningful contributions to the problem of spectral CT reconstruction. To accomplish this, we refine our previously proposed RSKR algorithm, reducing its computational complexity and its potential for introducing spectral bias, while maintaining highly robust performance and fast convergence at noise levels and material concentrations realistic for *in vivo* micro-CT imaging. Further regarding regularization, in this work we are among the first to demonstrate the integration of dictionary learning and sparse coding into a realistically sized, multi-dimensional (3D + energy) CT reconstruction problem. Specific to hybrid spectral CT reconstruction, we further develop our previously introduced algebraic framework [38], including substantial additional details on its derivation and dramatically improving the robustness of its regularization, making it applicable to realistic problems. Following from the digital and *in vivo* experiments presented in this work, we prove the value of our proposed methods through the largely unprecedented separation of realistic concentrations of iodine and barium, heavy-metal contrast materials with K-edge features separated by only 4.2 keV.

## Methods

In this investigation of hybrid CT reconstruction, we begin by outlining our proposed algebraic framework (*Algebraic reconstruction* sub-section). Using the split Bregman method, the algebraic reconstruction results are subject to penalties which enforce sparsity and low spectral rank (*Spectral regularization*). Leading to the practical application of hybrid reconstruction, technical details regarding our hybrid micro-CT system setup and our data acquisition and preprocessing strategy are then provided (*Dual-source*). Based on these details, a realistic simulation experiment is conducted to establish limits on the expectations of resolution transfer and contrast material decomposition accuracy possible with hybrid reconstruction (*Digital simulation*). Finally, details are provided for an *in vivo* hybrid micro-CT experiment using a mouse model of soft-tissue sarcoma (*In vivo experiment*).

### Algebraic reconstruction of hybrid CT data

#### Hybrid data fidelity

We employ an algebraic approach for hybrid, spectral CT reconstruction. The log-transformed, system-calibrated, and vectorized projection data is represented by $Y=[y_{e=1},\ldots ,y_{e=n_{e}}]$ for the PCD chain and by **w** for the EID chain, where $y_{e}\in R^{n_{y}\times 1}$ and $w\in R^{n_{w}\times 1}$. n_y_ and n_w_ equal the number of detector elements times the number of projections for each chain. n_e_ is the number of energy bins sampled with the PCD. The projection data is used to reconstruct $X_{L}=[x_{L,e=1},\ldots ,x_{L,e=n_{e}}]$, the low-column-rank EID data, and $X_{S}=[x_{S,e=1},\ldots ,x_{S,e=n_{e}}]$, the sparse, high-resolution spectral contrast ($x_{L},x_{S}\in R^{n_{x}\times 1}$). For simplicity, a single spectral data set from the EID chain is assumed (rank(X_L_) ≈ 1); however, we note that this is not generally required for reconstruction problems based on low rank and sparse decomposition.

Hybrid reconstruction is then performed via weighted least-squares optimization:

$$[X_{L},X_{S}]=\begin{array}{c} \arg\;min\\ X_{L},X_{S} \end{array}\sum_{e=1}^{n_{e}}\left[\frac{1}{2}{\left\|Rx_{L,e}-w\right\|}_{Q}^{2}+\frac{\lambda_{C,e}}{2}{\left\|AB(x_{L,e}+x_{S,e})-y_{e}\right\|}_{Z_{e}}^{2}\right].$$ (1)

The two least-squares terms enforce data fidelity relative to **w** and **y**_*e*_, respectively, for each energy bin sampled with the PCD detector. The fidelity terms are balanced by the energy-dependent scaling parameter, λ_C,*e*_ (vectorized form: **λ**_**C**_). Expanding the first data fidelity term, the ‖⋅‖_Q_ notation denotes least-squares weighting:

$${\left\|Rx_{L,e}-w\right\|}_{Q}^{2}≔{\left(Rx_{L,e}-w\right)}^{T}Q^{-1}(Rx_{L,e}-w).$$ (2)

R is the system projection matrix for the EID data ($R^{n_{w}\times n_{x}}$). As in [39], the least-squares weights are calibrated based on the observed projection data, as an estimate of the expected projection data, and the system-calibrated scaling parameters g and η:

$$Q=diag\left([q_{i=1}^{2},\ldots ,q_{i=n_{w}}^{2}]\right),$$ (3)

$$q_{i}^{2}=g_{i}\;\exp\left(w_{i}/\eta \right).$$ (4)

The diagonal elements of Q represent variance estimates for each line integral of the projection data. An analogous, energy-dependent weighting matrix, Z_*e*_, is constructed for each energy bin of the PCD chain. Notably, the use of diagonal weighting matrices approximates the detector noise as uncorrelated between neighboring detector pixels.

Returning to Eq 1, the following signal model is implied for the PCD data:

$$y_{e}=ABx_{e}+\varepsilon_{e},\;\varepsilon_{e,i}∼N(0,z_{e,i}^{2}),$$ (5)

$$x_{e}=x_{L,e}+x_{S,e}.$$ (6)

The high-resolution PCD data is related to the observed, low-resolution PCD data through the blurring and resampling operator, B ($R^{n_{x\prime }\times n_{x}}$), the PCD projection matrix, A ($R^{n_{y}\times n_{x\prime }}$), and additive, zero-mean Gaussian noise, **ε**_*e*_ ($R^{n_{y}\times 1}$). n_x′_ denotes the number of voxels in the low-resolution PCD data. Notably, the inclusion of Gaussian noise in the signal model approximates the Poisson photon statistics as Gaussian under log transformation and under the assumption of adequate photon flux in each energy bin of the PCD data. Consistent with the use of diagonal weighting matrices, the noise is assumed uncorrelated between detector elements at the native, low resolution.

Under the (somewhat liberal) assumption that the point-spread function for each imaging chain is well approximated with a spatially-invariant Gaussian kernel (G), the B operator resamples the high-spatial-resolution EID data (G_EID_) to match low-resolution PCD data (G_PCD_) with an appropriate resampling kernel (G_r_):

$$G_{r}(j-i)=\exp\left(-\frac{{\left\|j-i\right\|}_{2}^{2}}{2\sigma_{r}^{2}}\right).$$ (7)

The Cartesian spatial vectors **i** and **j** denote discrete sampling locations (voxel centers) within the high- (**x**_*e*_(**i**)) and low- ($x_{e}^{\prime }(j)$) spatial-resolution data, respectively. Note that we define the **x**_*e*_(**i**) notation to imply the treatment of vectors (**x**_*e*_) as volumes indexed by 3D coordinates (**i**). The appropriate standard deviation for the resampling kernel, σ_r_, is analytically computed from the measured full-width-at-half-maximum (FWHM) of the EID and PCD point-spread functions:

$$\sigma_{r}=\frac{\sqrt{{FWHM}_{PCD}^{2}-{FWHM}_{EID}^{2}}}{2\sqrt{2\;\ln\left(2\right)}}.$$ (8)

Resampling is then performed with a discrete, normalized convolution operation evaluated at each sampled spatial location within the low-resolution data, **j**:

$$x_{e}^{\prime }(j)=c\frac{\sum_{i}x_{e}(i)G_{r}(j-i)}{\sum_{i}G_{r}(j-i)}=B(j)x_{e},\;x_{e}^{\prime }=Bx_{e}$$ (9)

B(**j**) denotes the blur operator evaluated to produce a low-resolution sample at position **j**. As denoted elsewhere in the paper, B**x**_*e*_ denotes this resampling operation evaluated at all spatial positions, **j**. Assuming isotropic voxels, the scalar multiplier, c, scales attenuation measurements based on the voxel length ratio between the reconstructions of the PCD and EID data. The logical transpose of this resampling operation, which will be required in the following derivation, estimates a high-resolution sample at position **i** from the low-resolution samples:

$$x_{e}(i)=\frac{1}{c}\frac{\sum_{j}x_{e}^{\prime }(j)G_{r}(j-i)}{\sum_{j}G_{r}(j-i)}=B^{T}(i)x_{e}^{\prime }{,\;x}_{e}=B^{T}x_{e}^{\prime }$$ (10)

#### Penalized algebraic reconstruction with the split Bregman method

Given that algebraic deblurring is an ill-conditioned inverse problem, particularly at noise levels encountered in small animal micro-CT, robust regularization is required for successful hybrid reconstruction. Redefining the data fidelity terms (Eq 1) as a function of **x**_L,*e*_ and **x**_S,*e*_, f(**x**_L,*e*_,**x**_S,*e*_), we introduce rank and sparsity constraints on the solution to the hybrid reconstruction problem:

$$[X_{L},X_{S}]=\begin{array}{c} \arg\;min\\ X_{L},X_{S} \end{array}\sum_{e=1}^{n_{e}}f(x_{L,e},x_{S,e})+\lambda_{L}{\left\|X_{L}\right\|}_{*}+\lambda_{S}{\left\|X_{S}\right\|}_{BTV}.$$ (11)

‖X_L_‖_*_ denotes the nuclear norm, or the sum of singular values of the matrix X_L_, which is a convex proxy for column rank [40]. ‖X_S_‖_BTV_ denotes the bilateral total variation (BTV, [21, 41]) of X_S_ and is a weighted L^1^ norm which jointly enforces intensity gradient sparsity between the columns of X_S_. BTV is discussed further in a subsequent section.

Following several previous works [27, 37], we solve Eq 11 using the split Bregman method and the add-residual-back strategy. Specifically, we split Eq 11 into a series of equivalent sub-problems, each of which is more tractable than the original problem. The nuclear norm of X_L_ is first reduced:

$$D_{L}=\begin{array}{c} \arg\;min\\ D_{L} \end{array}\sum_{e=1}^{n_{e}}\frac{1}{2}{\left\|x_{L,e}+v_{L,e}-d_{L,e}\right\|}_{2}^{2}+\frac{\lambda_{L}}{\mu_{L}}{\left\|D_{L}\right\|}_{*},$$ (12)

where D_L_ (**d**_L,*e*_) and V_L_ (**v**_L,*e*_) are auxiliary variables introduced during the splitting which track the regularized estimate of X_L_ and the regularization residuals, respectively. Eq 12 can be solved via soft thresholding of the column- (energy-) wise singular values of X_L_ + V_L_ [40]. Second, the BTV of X_S_ is reduced with additional auxiliary variables D_S_ and V_S_:

$$D_{S}=\begin{array}{c} \arg\;min\\ D_{S} \end{array}\sum_{e=1}^{n_{e}}\frac{1}{2}{\left\|x_{S,e}+v_{S,e}-d_{S,e}\right\|}_{2}^{2}+\frac{\lambda_{S}}{\mu_{S}}{\left\|D_{S}\right\|}_{BTV}.$$ (13)

The cost in Eq 13 is reduced by the application of joint bilateral filtration (BF) between the columns of X_S_ + V_S_ [36].

Following from the add-residual-back strategy, the residual variables associated with each regularization term are then updated:

$$V_{L}=X_{L}+V_{L}-D_{L},$$ (14)

$$V_{S}=X_{S}+V_{S}-D_{S}.$$ (15)

Notably, the use of equality signs in Eqs 14 and 15 represents overwriting of the values in the V_L_ and V_S_ variables with the computed quantities. Finally, a lower-cost solution for the original cost function (Eq 11) is found by solving the following convex optimization problem for each energy:

$$[X_{L},X_{S}]=\begin{array}{c} \arg\;min\\ X_{L},X_{S} \end{array}\sum_{e=1}^{n_{e}}\left[f(x_{L,e},x_{S,e})+\begin{array}{c} \frac{\mu_{L,e}}{2}{\left\|x_{L,e}+v_{L,e}-d_{L,e}\right\|}_{2}^{2}+{\frac{\mu_{S,e}}{2}\|x_{S,e}+v_{S,e}-d_{S,e}\|}_{2}^{2} \end{array}\right].$$ (16)

Notably, in Eq 16 we have introduced energy dependence for the regularization parameters μ_L,*e*_ and μ_S,*e*_. These parameters are scaled with energy to account for differences in attenuation magnitude and noise level (see below). The λ_L_ and λ_S_ parameters are also, technically, energy dependent (Eqs 12 and 13); however, their effective values are adapted based on the input data (X_L_ + V_L_, X_S_ + V_S_; see the *Spectral regularization* sub-section), and they are not explicitly computed or assigned.

Because Eq 16 is convex and evaluated separately for each energy, it can be solved for each energy following differentiation with respect to **x**_L,*e*_ and **x**_S,*e*_:

$$R^{T}Q^{-1}Rx_{L,e}+\lambda_{C,e}B^{T}A^{T}Z_{e}^{-1}AB(x_{L,e}+x_{S,e})+\mu_{L,e}x_{L,e}=R^{T}Q^{-1}w+\lambda_{C,e}B^{T}A^{T}Z_{e}^{-1}y_{e}+\mu_{L,e}(d_{L,e}-v_{L,e}),$$ (17)

$$\lambda_{C,e}B^{T}A^{T}Z_{e}^{-1}AB(x_{L,e}+x_{S,e})+\mu_{S,e}x_{S,e}=\lambda_{C,e}B^{T}A^{T}Z_{e}^{-1}y_{e}+\mu_{S,e}(d_{S,e}-v_{S,e}).$$ (18)

Due to the large size of the projection operators in these linear equations (R, A; corresponding backprojection operators, R^T^, A^T^), we iteratively solve Eqs 17 and 18 for **x**_L,*e*_ and **x**_S,*e*_ using the biconjugate gradient stabilized method (BiCGSTAB, [42]). We have successfully employed BiCGSTAB for regularized algebraic reconstruction in previous work [36]. Multiple Bregman iterations (evaluations of Eqs 12 and 16) are typically required to achieve convergence with respect to the original cost function (Eq 11). The algorithm is terminated when a maximum number of iterations is reached or when the change in the magnitude of the residual variables between iterations falls below some tolerance.

One primary difficulty in finding meaningful solutions to Eq 11 is the selection of the regularization parameters λ_C,*e*_, μ_L,*e*_, and μ_S,*e*_ (Eqs 12–16), all of which depend on energy. The λ_C,*e*_ parameter for each energy balances the EID data fidelity term with the corresponding PCD data fidelity term. For the experiments presented in this work, the field-of-view coverage and total attenuation were similar enough that λ_C,*e*_ = 1 made a reasonable choice for all energies. Selection of the μ_L,*e*_ and μ_S,*e*_ parameters for each energy, which balance the data fidelity and regularization terms of the cost function following splitting (Eq 16), was more complicated due to significant differences in noise variance between the energy bins of the PCD data. Assuming approximate spatial uniformity of the noise level within each reconstructed energy bin, the image domain noise standard deviation can be robustly estimated as the median absolute deviation (MAD) of the component of the redundant Haar wavelet transform high-pass filtered along each dimension (e.g. in 3D: HHH) [43]:

$$\sigma_{e}=MAD(x_{e})=1.4826*median(\left|HHH(x_{e})\right|).$$ (19)

Practically, we note that the accuracy of this noise estimation scheme need only be relative to other energies, given that the noise estimates are only used as ratios (see below) or after multiplication with a user-specified regularization parameter (see *Spectral regularization*). Furthermore, the requirement of a spatially uniform noise level is relaxed when using weighted least-squares (Eq 2), since low data-fidelity weights are assigned to the nosiest (largest magnitude) line integrals. This increases the effective regularization strength in highly attenuating features.

Given MAD-based noise estimates for each energy, μ_L,*e*_ and μ_S,*e*_ are then calibrated with standard deviation ratios, δ_*e*_, and with an empirically determined scaling factor, α:

$$\delta_{e}=\frac{\left(\sigma_{e}/\mu_{water,e}\right)}{\min_{e}\left(\sigma_{e}/\mu_{water,e}\right)},$$ (20)

α∈[0.001,0.01]. (21)

The notation min_*e*_ denotes that each noise level estimate is normalized relative to the smallest estimate measured over all energies. Normalization of σ_*e*_ by the expected attenuation of water in each energy bin, μ_water,*e*_, promotes consistent noise levels when the final reconstructed results are converted to the Hounsfield scale or are used for material decomposition. The convenience of achieving good reconstruction results with a predetermined α parameter (Eq 21) and the rapid convergence of our overall algorithm rely on further internal adaptation of the regularization strength (see *Spectral regularization*). Taking inspiration from the right-hand sides of Eqs 17 and 18 and scaling with respect to these parameters (α, δ_*e*_), meaningful values of μ_L,*e*_ and μ_S,*e*_ are analytically estimated with the following formulas:

$$\mu_{L,e}=\alpha \delta_{e}\frac{{\left\|R^{T}Q^{-1}w+\lambda_{C,e}B^{T}A^{T}Z_{e}^{-1}y_{e}\right\|}_{2}}{{\left\|x_{L,e}\right\|}_{2}},$$ (22)

$$\mu_{S,e}=\alpha \delta_{e}\frac{{\left\|\lambda_{C,e}B^{T}A^{T}Z_{e}^{-1}y_{e}\right\|}_{2}}{{\left\|x_{S,e}\right\|}_{2}}.$$ (23)

**Fig 1:**
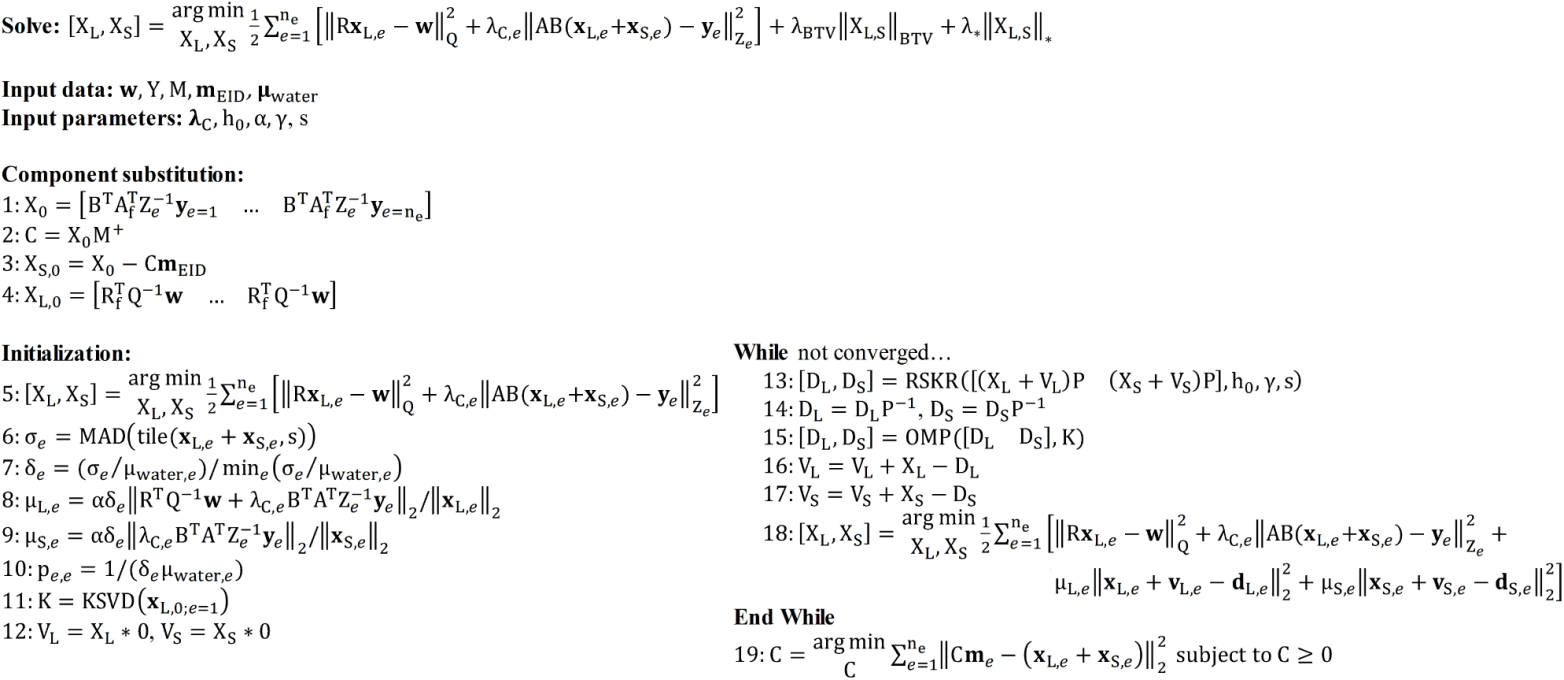
Pseudocode for hybrid CT reconstruction with the split Bregman method. The objective of hybrid CT reconstruction is to synthesize high-resolution, spectral CT reconstructions from high-resolution, energy-integrated projection data, **w**, and lower-resolution and nosier photon-counted projection data, Y. In addition to the projection data, expected material sensitivity values (M, **m**_**EID**_) and user-specified regularization parameters (**λ**_**C**_, h_0_, α, γ, s) are provided as inputs. FBP reconstruction and component substitution provide estimates of the reconstructed results (steps 1–4) which are further refined with algebraic reconstruction during initialization (steps 5–12). Following initialization, the hybrid reconstruction results are refined under low spectral rank and intensity-gradient sparsity constraints through iterative application of the split Bregman method (step 13–18). Following reconstruction, the hybrid results are used to compute high-resolution material decompositions (step 19).

Following modifications described in the *Spectral regularization* sub-section, Fig 1 summarizes our application of the split Bregman method to the problem of hybrid CT reconstruction.

#### Component substitution and material decomposition

As shown in Fig 1, the first steps (1–4) of hybrid reconstruction initialize X_L_ (X_L,0_) and X_S_ (X_S,0_). These component substitution steps are, themselves, initialized with filtered backprojection (FBP) reconstructions. Specifically, for the experiments in this work we used the FDK algorithm [44] with a ramp filter. FBP reconstruction (denoted $A_{f}^{T}$) of each column (energy) of Y followed by upsampling (B^T^) yields an estimate of the high-resolution PCD data at each energy:

$$X_{0}=[\begin{array}{ccc} B^{T}A_{f}^{T}Z_{e}^{-1}y_{e=1} & \ldots & B^{T}A_{f}^{T}Z_{e}^{-1}y_{e=n_{e}} \end{array}].$$ (24)

Nominally, X_S,0_ can then be estimated as the difference between X_0_ and X_L,0_. In practice, however, due to initial resolution differences between X_0_ and X_L,0_, this strategy was found to introduce high frequency artifacts which were not well addressed by subsequent regularization. Therefore, following from our previous work [7, 14], we instead used material decomposition to estimate X_L,0_ from X_0_ and then estimated X_S,0_ as the difference between these resolution-matched estimates:

$$C=XM^{+},$$ (25)

$$X_{S,0}=X_{0}-Cm_{EID}.$$ (26)

The material sensitivity matrix for the PCD data, M ($R^{n_{m}\times n_{e}}$), relates the attenuation at each energy to material specific measurements (e.g. m_I,*e* = 1_ is the attenuation of 1 mg/mL of iodine at energy 1; n_m_, total number of basis materials). Inversion (n_e_ = n_m_) or, more generally, pseudo-inversion (n_e_ > n_m_) of the material sensitivity matrix (M^+^) is used to estimate the material concentrations (fractions) C ($R^{n_{x}\times n_{m}}$; Eq 25). Multiplication of these concentrations by the material sensitivities measured in the EID data, **m**_EID_ ($R^{n_{m}\times 1}$), yields the required low-resolution estimate for X_L,0_. Following from the *Introduction*, we note this is a form of component substitution, a common strategy for pan-sharpening, given that X_S_ will be added to the high-resolution estimate for X_L,0_ produced from the EID projection data (step 4). This estimation and substitution procedure is used to condition and accelerate the convergence of the initial algebraic reconstruction (initialization; Fig 1, step 5) prior to regularized reconstruction with the split Bregman method.

Following hybrid reconstruction with the split Bregman method, the final resolution-enhanced material maps, C, are obtained via material decomposition subject to a non-negativity constraint (Fig 1, step 19):

$$C=\begin{array}{c} \arg\;min\\ C \end{array}\sum_{e=1}^{n_{e}}{\left\|Cm_{e}-(x_{L,e}+x_{S,e})\right\|}_{2}^{2}\;subject\;to\;C\geq 0.$$ (27)

As before, this problem is readily solved by pseudo-inversion of the material sensitivity matrix, M (columns: **m**_*e*_). Non-negativity is enforced on the least-squares decomposition of each voxel by orthogonal projection onto the subspace of positive material concentrations [45]. Building on the approach of Alvarez and Macovski [46], we choose the photoelectric effect (PE), Compton scattering (CS), iodine (I), and barium (Ba) as material basis functions in this work. Notably, the K-edges of iodine (33.2 keV) and barium (37.4 keV), the primary features which discriminate their attenuation from the PE and CS basis functions, are less than 5 keV apart, providing a rigorous test for the regularization strategy we propose in the next sub-section.

### Spectral regularization

In this sub-section we provide a mathematical formalism for our regularization strategies: rank-sparse kernel regression (RSKR) and dictionary learning and sparse coding. Specifically, we provide details regarding our implementation of each algorithm, and we establish their relationship to rank-sparsity constrained hybrid spectral CT reconstruction.

#### Joint bilateral filtration and bilateral total variation

RSKR is based on the application of joint bilateral filtration (BF) to spectral CT data. Under the assumption of structural redundancy between energies, joint BF performs non-linear, edge-preserving smoothing to enforce matching intensity gradient sparsity patterns between energies [7]. Mathematically, we apply joint BF as follows:

$$BF(x_{e}(o))=\frac{\sum_{p}D(p)R_{joint}(o,p)(\Delta_{K}x_{e}(o,p))}{\sum_{p}D(p)R_{joint}(o,p)},$$ (28)

$$R_{joint}(o,p)=exp\left(-\frac{1}{2}\sum_{e=1}^{n_{e}}\frac{{\left(\Delta_{K}x_{e}(o,p)\right)}^{2}}{{\left(h_{e}\sigma_{e}\right)}^{2}}\right),$$ (29)

$$D(p)=\left\{ \begin{array}{l} 1,\;{\left\|p\right\|}_{2}\leq b\\ 0,\;{\left\|p\right\|}_{2}>b \end{array}\right..$$ (30)

The Cartesian spatial vectors **o** and **p** denote the voxel being filtered and the set of all discrete spatial offsets within the filtration neighborhood, respectively. BF then takes the form of a 3D convolution operation (Eq 28) which adapts to the local filtration domain, D(**p**) (b, domain radius; Eq 30), with jointly computed range weights, R_joint_(**o**,**p**) (Eq 29). The contribution of each energy to the joint range kernel is scaled by the noise level measured immediately prior to filtration (σ_*e*_, Eq 29; measured as the MAD, Eq 19) and a scalar parameter which controls regularization strength, h_*e*_. The jointly Gaussian range weights are then computed from the intensity differences measured within the filtration domain:

$$\Delta_{K}x_{e}(o,p)≔x_{e}(o-p)-\sum_{t}K(t)x_{e}(o-t).$$ (31)

K(**t**) is a spatial resampling kernel (domain indexed by **t**) which improves robustness in band-limited data and ensures consistent regularization results at edges. In most of our previous work, we have used a second-order resampling kernel for K(**t**) [7]. Here, however, we use a delta function:

$$K(t)=\left\{ \begin{array}{l} 1,\;t=0\\ 0,\;t\neq 0 \end{array}\right.,$$ (32)

where **0** denotes the Cartesian spatial position of the kernel’s origin. The motivation for this change is for compatibility with a tiling operation we introduce (see below) and to avoid unnecessary resolution losses prior to the application of sparse coding (see *Dictionary learning*).

Comparing this implementation of joint BF with more common, direct sparsity constraints (e.g. TV), joint BF improves robustness because it operates on multiple scales of image derivatives. Furthermore, the abstraction of gradient information to intensity-independent probabilities enables contrast-independent spectral smoothing, while the adjustment of regularization strength and range kernel contribution based on the noise level measured at each energy adapts to the specific problem. These properties of joint BF are encapsulated in the BTV metric. Specifically, within the context of gradient-sparsity constrained, iterative reconstruction and the split Bregman method, BTV is measured as the application of the joint BF weights to the intensity gradients measured for each energy [21, 41]:

$${\frac{\lambda_{S}}{\mu_{S}}\|D_{S}\|}_{BTV}≔\sum_{e=1}^{n_{e}}\frac{\lambda_{S,e}}{\mu_{S,e}}\sum_{o}\left|\frac{\sum_{p}D(p)R_{joint}(o,p)(\Delta_{K}d_{S,e}(o,p))}{\sum_{p}D(p)R_{joint}(o,p)}\right|.$$ (33)

The application of BF to each column of X_S_ + V_S_ using the jointly computed range weights reduces BTV, yielding a lower-cost solution for D_S_ in the regularization update step (Eq 13).

**Fig 2:**
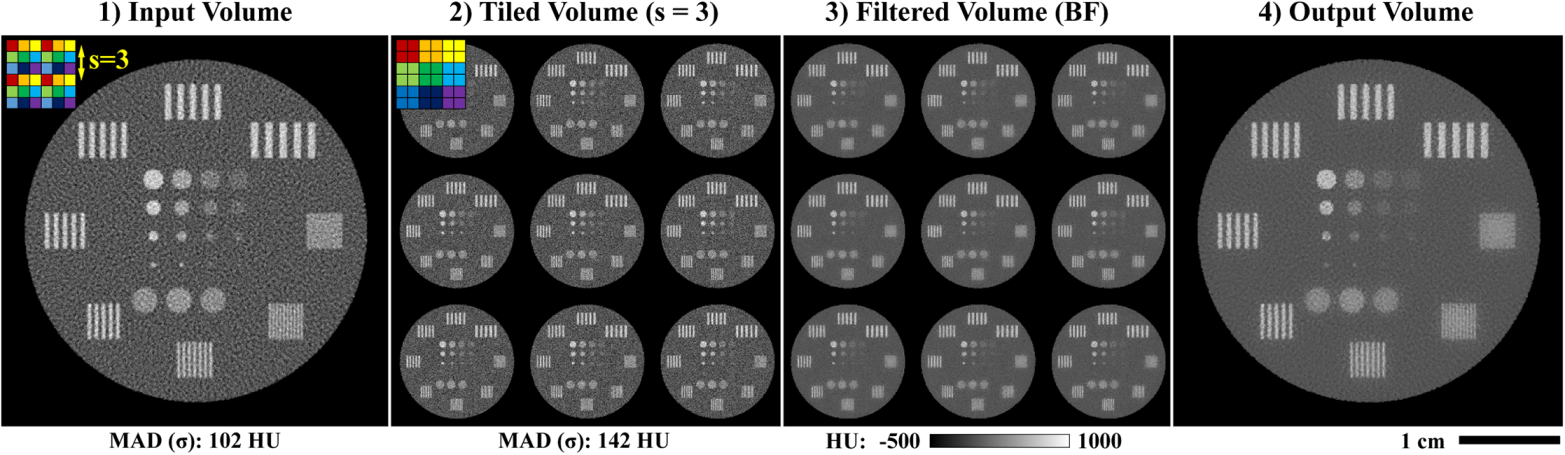
Bilateral filtration (BF) with tiling. Given a volume to be filtered (1), a tiling operation is first applied to reduce correlation in the noise and to effectively extend the filtration domain size (2). BF is applied to this tiled volume (3), and then the output volume is recovered by reversing the tiling operation (detiling, 4). Note the difference in the median absolute deviation (MAD) measured in the input (1) and tiled (2) volumes (bottom), given an input volume which has been upsampled (B^T^ operator). As with all figures in this work, the window width and level for each panel as well as the appropriate units are as indicated by the calibration bar (3, bottom; HU: Hounsfield units). The absolute scale is as shown by the scale bar (4, bottom right). All CT reconstructions and material decompositions in this work are presented as single 2D slices, without averaging between slices, unless otherwise specified.

Given this formalism for joint BF and BTV minimization, we note that BF performs sub-optimally in removing low-frequency, correlated noise which is introduced when upsampling noisy, low-resolution PCD data (B^T^ operator). Low frequency denoising performance of BF could be addressed by increasing the diameter of the filtration domain (i.e. b > 6, our usual value; Eq 30); however, computation time scales cubically with kernel diameter. Instead, as previously mentioned in the *Introduction*, we take inspiration from the problem of super-resolution, constructing several aliased volumes from each input volume (energy) prior to noise estimation with the MAD and to the application of joint BF. Given the appearance of these aliased volumes when they are constructed within the original volume (Fig 2), we call this operation tiling. Practically, tiling works well for several reasons. First, when a delta function is used for resampling (Eq 32), BF operates on the intensity of individual voxels. Combining this property with the high degree of redundancy of x-ray CT attenuation values, BF generally performs well when applied to tiled volumes. Low-frequency noise in the original volume appears as high-frequency noise in the tiled volume, more accurately reflecting the amount of noise that must be removed to match the spatial fidelity of the EID data and more accurately adapting the regularization strength between iterations of the reconstruction algorithm. The tiling operation itself negligibly increases computation time, since it involves a simple remapping of voxel spatial locations (see below), followed by BF of the tiled volume with a usual domain size (here, b = 6). The end effect is that tiled BF tends to quantize local intensity values to match global intensity modes, readily addressing much lower frequency noise than standard BF [38]. As illustrated in the *Simulations* sub-section of the *Results*, the potential downside to tiled BF is that intensity bias must be carefully managed to avoid compromising material decomposition fidelity.

The tiling operation is implemented as a function of the stride parameter, s, which controls the step size between voxels to be included within the same tiled sub-volume. Intuitively, s should be larger than the FWHM of the resampling kernel used to upsample the low-resolution data (measured in high-resolution voxel widths; here, s = 3 voxels is used). Using this stride, the tiling operation maps intensity values at Euclidean voxel indices within the original volume, **m** (intensities **x**(**m**)), to tiled voxel indices, **n** (intensities **t**(**n**)), within a constant, global coordinate system:

t(n)=x(m). (34)

The 3D index vectors are themselves indexed by *o*, denoting the x (*o* = 0), y (*o* = 1), and z (*o* = 2) axes, respectively. The number of voxels along each axis is contained within the vector **g** (padded to integer multiples of s):

$$m_{o},\;n_{o}\;\in [0,g_{o}-1].$$ (35)

Consecutive integer indices are assigned to each axis of **n**:

$$n_{o}≔[\begin{array}{cccc} 0 & 1 & \ldots & g_{o}-1 \end{array}].$$ (36)

Given these definitions, the following coordinate transformation can then be used with Eq 34 to map the input volume intensities to the tiled volume intensities:

$$m_{o}=s*mod\left(n_{o},\frac{g_{o}}{s}\right)+\left\lfloor n_{o}*\frac{s}{g_{o}}\right\rfloor .$$ (37)

The mod(*a*,*b*) function denotes the modulo operator applied to *a* and *b*, and ⌊⋅⌋ denotes rounding down to the nearest integer. Following BF and reusing the initial **m** and **n** indices, the tiling operation is then reversed with the following inverse mapping operation (detiling):

x(m)=t(n). (38)

The tiling and detiling operations and their relation to BF are represented graphically in Fig 2.

#### RSKR: Joint bilateral filtration of singular vectors

Within the context of the split Bregman method and hybrid spectral CT reconstruction, the dual objective of RSKR is to enforce matching intensity-gradient sparsity patterns and low rank between the EID and PCD reconstructions. To develop this dual rank-sparsity constraint, we first note that the constraint terms of the objective function (Eq 11), which are based on a general model for low rank and sparse matrix decomposition (i.e. robust principal component analysis, [34]), can be further specialized for the problem of hybrid spectral CT reconstruction. Specifically, Eq 11 enforces low rank between the columns of X_L_ and matching intensity gradient sparsity patterns between the columns of X_S_; however, it does not exploit the fact that X_L_ and X_S_ must exhibit complementary intensity gradient sparsity patterns (i.e. they must add to the PCD data). Also, it does not exploit that the rank of X_L_ + X_S_ should ideally be lower than the number of columns when the number of sampled energies exceeds the number of unique materials (n_m_ < n_e_). To incorporate these additional constraints, we replace Eqs 12 and 13 with the following combined update equation:

$$D_{L,S}=\begin{array}{c} \arg\;min\\ D_{L,S} \end{array}\frac{1}{2}\sum_{i=1}^{{2n}_{e}}{\left\|x_{L,S,i}+v_{L,S,i}-d_{L,S,i}\right\|}_{2}^{2}+\lambda_{BTV}{\left\|D_{L,S}\right\|}_{BTV}+\lambda_{*}{\left\|D_{L,S}\right\|}_{*},$$ (39)

$$D_{L,S}=[\begin{array}{cc} D_{L} & D_{S} \end{array}],\;X_{L,S}=[\begin{array}{cc} X_{L} & X_{S} \end{array}],\;V_{L,S}=[\begin{array}{cc} V_{L} & V_{S} \end{array}].$$ (40)

The bracket notation ([· ·]) denotes concatenation of the columns of each component matrix (Eq 40). As in Eqs 12 and 13, the new regularization parameters, λ_BTV_ and λ_*_, have some energy dependence; however, they are never explicitly computed or set (see below).

Comparing Eq 39 with the original objective function (Eq 11), the dual constraint terms appear counter-productive given the apparent difficulty in simultaneously reducing BTV and the nuclear norm subject to a data fidelity constraint. To alleviate this difficulty and to enforce matching intensity gradient sparsity patterns and low rank between the columns of X_L,S_ + V_L,S_, we first perform a weighted, reduced singular value decomposition:

$${\text{p}}_{e,e}=1/\left(\delta_{e}\mu_{\text{water},e}\right),$$ (41)

$$\left[{\text{U}}_{0},{\text{E}}_{0},{\text{V}}_{0}\right]=\text{SVD}\left(\left[\begin{array}{cc} \left({\text{X}}_{\text{L}}+{\text{V}}_{\text{L}}\right)\text{P} & \left({\text{X}}_{\text{S}}+{\text{V}}_{\text{S}}\right) \end{array}\text{P}\right]\right).$$ (42)

The diagonal weighting matrix, P, (Eq 41; scalar diagonal elements p_*e*,*e*_) is a function of the standard deviation ratios computed during the initialization of the algorithm, δ_*e*_, (Eq 20) and the expected attenuation of water at each energy, μ_water,*e*_. We call this weighting scheme prioritization because it encourages the most significant singular vectors, U_0_, (i.e. the singular vectors with the largest associated singular values, E_0_) to correspond with significant modes of spectral contrast rather than noise. Specifically, the water normalization roughly equalizes the attenuation magnitude at each energy, up to significant spectral differences (e.g. changes in attenuation over K-edges). The δ_*e*_ weighting factors, which are based on water-normalized noise measurements (Eq 20), further bias the most significant singular vectors to best fit the energies with the highest signal-to-noise ratios.

Given the prioritized singular value decomposition of the input data, we then solve for denoised singular vectors, U (n_u_ columns), under a BTV constraint:

$$U=\begin{array}{c} \arg min\\ U \end{array}\frac{1}{2}\sum_{i=1}^{n_{u}}{\left\|u_{i}-u_{0,i}\right\|}_{2}^{2}+\lambda_{BTV}{\left\|U\right\|}_{BTV}.$$ (43)

**Fig 3:**
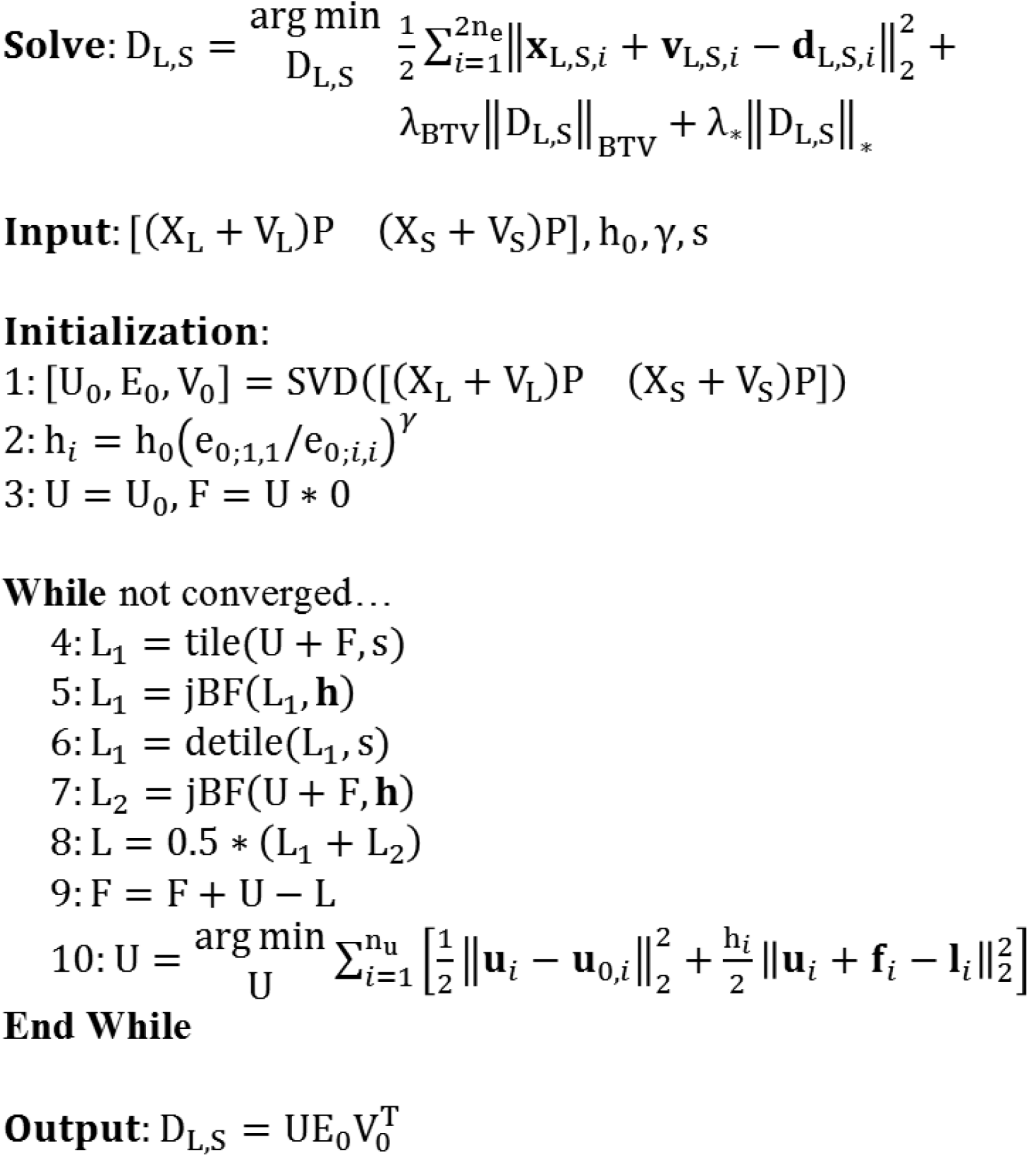
Pseudocode for regularization with rank-sparse kernel regression (RSKR). The objective of RSKR is to enforce matching intensity gradient sparsity patterns and low column rank on a matrix of spectral CT data taken as input. To achieve this, RSKR operates on a weighted singular value decomposition of the spectral data (step 1) and calibrates the regularization strength for each singular vector based on ratios of the corresponding singular values (step 2). Intensity gradient sparsity patterns are copied between singular vectors through joint bilateral filtration (jBF; steps 5 and 7). A rank reduction effect is achieved by allowing proportionally stronger regularization for less significant singular vectors (step 2, step 10) over the course of several internal Bregman iterations. Within the context of hybrid spectral CT reconstruction, RSKR is embedded as a sub-step within our proposed algorithm (Fig 1, step 13).

In terms of the pseudocode for our algorithm, we refer to the process of performing singular value decomposition and then solving Eq 43 as RSKR (Fig 3). Within a single step of a global Bregman iteration (Fig 1, step 13), we solve Eq 43 using several internal Bregman iterations and an independent set of variables which are reset between global iterations (Fig 3):

$$L=\begin{array}{c} \arg min\\ L \end{array}\frac{1}{2}\sum_{i=1}^{n_{u}}{\left\|u_{i}+f_{i}-l_{i}\right\|}_{2}^{2}+\lambda_{h}{\left\|L\right\|}_{BTV},$$ (44)

F=F+U−L, (45)

$$U=\begin{array}{c} \arg min\\ U \end{array}\sum_{i=1}^{n_{u}}\left[\frac{1}{2}{\left\|u_{i}-u_{0,i}\right\|}_{2}^{2}+\frac{h_{i}}{2}{\left\|u_{i}+f_{i}-l_{i}\right\|}_{2}^{2}\right].$$ (46)

As before (Eq 13), the cost associated with Eq 44 is reduced through the application of joint BF to the columns of U + F, including tiling and noise estimation with the MAD prior to filtration, yielding the regularized output, L (analogous to D in the global Bregman iterations). Eq 45 updates the regularization residuals stored in F (analogous to V in the global Bregman iterations). The data fidelity update equation (Eq 46) is convex and can be solved analytically for each singular vector following differentiation with respect to **u**_*i*_:

$$u_{i}=\frac{u_{0,i}+h_{i}(l_{i}-f_{i})}{1+h_{i}}.$$ (47)

In the simulation and *in vivo* experiments detailed later in this work, 3–6 Bregman iterations were required for convergence of RSKR (<1% change in the regularization residual magnitude between iterations). After convergence, the regularized estimate of the input data, D_L,S_, is recovered using the regularized singular vectors, U:

$$D_{L,S}=[\begin{array}{cc} D_{L} & D_{S} \end{array}]=UE_{0}V_{0}^{T}.$$ (48)

The relative magnitudes of the columns of D_L_ and D_S_ are then recovered using the priority weights (Fig 1, step 14):

$$D_{L}=D_{L}P^{-1},\;D_{S}=D_{S}P^{-1}.$$ (49)

From the description of RSKR, it is not immediately clear how we achieve rank reduction to enforce structural redundancy between the columns of X_L_ and X_S_. Perhaps the most direct method would be to apply soft thresholding to the singular values (E_0_, [40]) in addition to denoising the singular vectors. This idea is particularly attractive since it, ostensibly, addresses the difficulty in reducing the cost associated with the dual rank-sparsity constraint directly (Eq 39). Practically, however, we have found that thresholding singular values based on a static threshold strikes a difficult balance between performing many iterations with a small threshold, which exacerbates computation for realistically large problems, and accepting a certain level of spectral bias with a larger threshold, which exacerbates errors in bias-sensitive material decompositions. An alternative approach for rank reduction that we used in previous work [35] replaces Eq 48 with the following reprojection step to yield the regularized results, [D_L_ D_S_]:

$$[\begin{array}{cc} D_{L} & D_{S} \end{array}]=U(U^{T}[\begin{array}{cc} (X_{L}+V_{L})P & \left(X_{S}+V_{S}\right) \end{array}P]).$$ (50)

Under the assumption that orthogonality is approximately maintained between the columns of U [47], this approach intrinsically reduces the nuclear norm as a function of the amount of noise removed from the columns of U, since U will necessarily deviate from the exact orthonormal subspace for the columns of [(X_L_ + V_L_)P (X_S_ + V_S_)P], U_0_. We believe this reprojection method will be highly effective in certain applications where some level of bias can be tolerated. Specifically, within the context of hybrid reconstruction problems, this reprojection step appears to provide some level of tolerance to missing data, cone-beam artifacts due to differences in magnification, and to more dramatic resolution differences than we deal with in this work (see [38]). In this work, however, we strictly use the more conservative method to produce the regularized results (Eq 48) because the spectral separation of iodine and barium is very sensitive to spectral bias (see *Contrast and resolution phantom*).

Regardless of which equation is used to produce the final, regularized results, apparent rank reduction is also achieved through manipulation of the scalar noise multiplier parameters used to scale the contribution of each singular vector to the jointly constructed range weights, h_*i*_ (h_*e*_, Eq 29). Rather than manually choosing values for each h_*i*_, however, we choose a single value, h_0_, which works well for most problems (h_0_ ≈ 1–2), and then we scale h_0_ based on ratios of the singular values and on a scaling parameter, *γ* (*γ* ≈ 0.5; Fig 3, step 2):

$$h_{i}=h_{0}{\left(e_{0;1,1}/e_{0;i,i}\right)}^{\gamma }.$$ (51)

The double subscript notation, e_0;*i*,*i*_, refers to the scalar diagonal elements of E_0_ starting from 1,1 for the first singular value. Notably, this scaling function is closely related to threshold scaling which has been proposed for non-convex soft thresholding of singular values [32]. Using the scaled h_*i*_ parameters, joint BF of the singular vectors (jBF(·); Fig 3, steps 5 and 7) places greater emphasis on preserving edge features in the most significant singular vectors which have been prioritized to contain comparatively lower levels of noise. When these same multiplier parameters are also used to control the balance between regularization and data fidelity in the data fidelity update step (Eq 46; Fig 3, step 10), RSKR has the effect of smoothing less significant singular vectors to match the image structure in more significant singular vectors over the course of multiple internal Bregman iterations.

Within the context of hybrid spectral CT reconstruction, where copies of the EID data make up one half of the inputs into RSKR (Fig 1, step 13), the most significant singular vectors are dominated by the EID data. This produces a unique regularization paradigm which balances between two extremes. In the one extreme (large *γ*), the joint BF kernel is entirely determined by the first singular vector, smoothing all other singular vectors to closely match the structure in the EID data. This has the benefit of copying high-frequency edge information from the EID data to the PCD data, potentially enhancing resolution, but may over-smooth spectral features which have low contrast in the EID data. This approach may also introduce significant bias between the regularized PCD reconstructions and the PCD projection data. At the other extreme (small *γ*), all singular vectors contribute equally to range kernel construction, preventing efficient regularization due to substantial noise is the less significant singular vectors. Choosing an intermediate value for *γ* (here, 0.5), provides a compromise between copying high frequencies from the EID data to the PCD data, while managing bias. During later applications of RSKR (later global Bregman iterations), when the noise level has equalized between the EID and PCD data, over-smoothing of high contrast features in the PCD data is prevented due to the non-trivial contribution of less significant singular vectors to the joint range kernel.

#### Dictionary learning and sparse coding

As we will show, the algebraic model we have proposed for hybrid spectral CT reconstruction successfully synergizes the reconstruction of EID and PCD data, yielding improved denoising performance and evidence of resolution enhancement. However, there is a subtle limitation to enforcing agreement between the EID and PCD reconstructions under L^2^ constraints (Eq 1): some amount of correlated noise and blurring will always bleed back into the EID reconstructions. This trade-off can be managed through the **λ**_C_ regularization parameters; however, relaxing the data fidelity constraint on the PCD data will generally lead to spectral bias. Supplementing the objective function with a rank-sparsity constraint enforced with RSKR provides some robustness to this blurring and correlated noise through joint filtration, which favors the EID data, and through tiling, which improves the removal of low frequency noise. However, as previously discussed, the PCD data cannot be ignored during the construction of the joint range kernel because of the risk of over-smoothing features with low spectral contrast in the EID data. A related issue is that the piece-wise constant image structure enforced by joint BF imperfectly matches the band-limited (smooth) edge features in both the EID and PCD data, bounding the performance of resolution enhancement.

To address these limitations, we adapt regularization with dictionary learning and sparse coding to the problem of hybrid CT reconstruction. Specifically, for dictionary learning we employ the K-SVD algorithm ([48], KSVD-Box v13), and for 3D sparse coding we employ our own GPU-based implementation of batch orthogonal matching pursuit (OMP) using progressive Cholesky factorization [49]. In hybrid reconstruction, we apply dictionary learning to the FBP reconstruction of the EID data (**x**_L,0;*e* = 1_) during the initialization phase (Fig 1, step 11; KSVD(·)) to minimize the following objective function [48]:

$$[\begin{array}{c} K,\Phi \end{array}]=\begin{array}{c} \arg min\\ K,\Phi \end{array}{\left\|X_{L,P}-K\Phi \right\|}_{F}^{2}\;subject\;to\;\forall i\;{\left\|\phi_{i}\right\|}_{0}\leq n_{0}.$$ (52)

$$K\in R^{n_{v}\times n_{a}},\Phi \in R^{n_{a}\times n_{p}},X_{L,p}\in R^{n_{v}\times n_{p}}.$$ (53)

X_L,P_ is a matrix of mean-subtracted, radial patches extracted from **x**_L,0;*e* = 1_ (n_v_: number of voxels per radial patch; n_p_: number of patches). The K-SVD algorithm approximates these patches as sparse, linear combinations of atoms (coefficient matrix: Φ; ‖⋅‖_0_: number of non-zero entries) drawn from an overcomplete dictionary, K (unit norm, zero-mean, column vectors; n_a_: total number of atoms). In more detail, the K-SVD algorithm alternates between two phases: (1) sparse coding (with OMP), to choose and update the non-zero coefficients of each column of Φ (n_0_: maximum allowed number of non-zero coefficients), and (2) dictionary updating, to improve the fidelity with which the dictionary atoms represent the training patches which use them. We leave most of the details of dictionary learning to the referenced work; however, we make note of several parameter choices. First, to learn the dictionary, we extracted overlapping, 3D, radial patches (domain defined as in Eq 30; b = 4) from the input volume and stored them as columns of the matrix X_L,P_. Specifically, 500,000 training patches with a variance higher than the noise level were extracted from all possible overlapping patches. The total number of dictionary atoms was chosen to be n_a_ = 1024 based on the heuristic criterion that the number of atoms should be four times larger than the number of voxels per patch [50]. The total number of K-SVD iterations used to learn the dictionary was 25 (one K-SVD iteration: one sparse coding phase, one dictionary updating phase). A maximum of n_0_ = 48 atoms with non-zero coefficients were allowed per patch during the sparse coding phase of the K-SVD algorithm; however, at convergence, far fewer atoms were required to meet the residual error criterion for most patches (indexed by *i*):

$$\varepsilon =n_{v}*{MAD\;(tile(x_{L,0;e=1},\;s))}^{2},$$ (54)

$${\left\|x_{L,P;i}-K\phi_{i}\right\|}_{2}^{2}\leq \varepsilon .$$ (55)

For the experiments in this work, the tile(·,s) function performed tiling as discussed in the *Joint bilateral filtration* section (stride, s = 3) for the purpose of robust estimation in the presence of low-frequency, correlated noise. With the exception of noise estimation, the dictionary learning and sparse coding operations were performed exclusively on untiled data. Additional details, such as the criterion for replacing under-used dictionary atoms and similar dictionary atoms, were handled with the default parameters in the referenced code.

Following convergence of the K-SVD algorithm (Fig 1, step 11), the resultant dictionary, K, is held fixed. Under the assumption that K provides a robust basis for representing new patches in related data sets, only sparse coding with OMP is performed for regularization with respect to the objective function (Eq 52). More specifically, OMP for regularization (OMP(·,K) function; Fig 1, step 15) involves several sub-steps: (1) noise estimation to calibrate the residual convergence criterion (Eq 55); (2) extraction of all overlapping radial patches from the input volume; (3) subtraction of the mean intensity from each patch; (4) sparse coding subject to the objective function (Eq 52) and the residual convergence criterion (Eq 55); (5) addition of the original patch mean back to each coded patch; and (6) recovery of the regularized volume through averaging of the overlapping patches. Our hybrid reconstruction algorithm preforms sparse coding with OMP on each column of [D_L_ D_S_] independently following RSKR (Fig 1, step 15). For regularization, the number of non-zero coefficients (# of atoms used to code each patch) was determined by the minimum number of atoms required to satisfy the residual error criterion, up to a maximum of n_0_ = 5 atoms with non-zero coefficients.

Returning to our motivations for incorporating dictionary learning and sparse coding into the hybrid reconstruction algorithm, a dictionary learned from a FBP reconstruction of the EID data encodes contrast-independent prior information about the signal characteristics which are expected in the final hybrid reconstruction results. Interestingly, there is little risk of overfitting the EID data during dictionary learning since we expect to recover nearly identical image structures in the results of hybrid reconstruction. Furthermore, because the dictionary is trained using the EID data only, any bias introduced during sparse coding with OMP will ideally make the regularized results more similar to the expected results. Finally, because the mean of each patch is strictly preserved and because the input to sparse coding should have a relatively low level of noise following RSKR, the risk of introducing spectral bias is extremely low.

#### Rank-sparsity constrained hybrid spectral CT reconstruction

Now that we have detailed the individual components of our proposed hybrid spectral CT reconstruction algorithm, we briefly summarize how its components fit together into a coherent algorithm. Following the pseudocode in Fig 1, the EID (**w**) and PCD (Y) projection data as well their expected material sensitivities (**m**_EID_ and M, respectively; also, **μ**_water_ in the PCD data) are provided as input. The user must specify several parameters (typical values) that influence the trade-off between EID and PCD data fidelity (**λ**_C_ = **1**), the amount of smoothing performed with joint BF (h_0_ ≈ 1–2), the overall trade-off between data fidelity and regularization (α ∈ [0.001,0.01]), the rank reduction effect of RSKR (*γ* ≈ 0.5), and the bias-variance trade-off in addressing low-frequency noise (s = 3). Initial estimates for X_L_ (X_L,0_), the redundant reconstructions of the EID data, and X_S_ (X_S,0_), the spectral contrast contributed by the PCD data, are produced via material decomposition and component substitution (steps 1–4). Prior to regularized iterative reconstruction, several quantities must be initialized (steps 5–12). Specifically, algebraic reconstruction with weighted least-squares is performed to satisfy the hybrid data fidelity constraints and to pre-condition the regularized portion of the algorithm for faster convergence (step 5). The reconstructed noise level of the data at each energy, σ_*e*_, is then estimated using tiling operations and MAD computations (step 6). Water-normalized ratios of these noise estimates, δ_*e*_, (step 7) are used to calibrate the regularization parameters, μ_L,*e*_ and μ_S,*e*_, (steps 8–9) as well as the SVD prioritization weights, p_*e*,*e*_, (step 10) for each energy, *e*. Independently, a dictionary of characteristic atoms, K, is learned using a FBP reconstruction of the EID data, **x**_L,0;*e* = 1_, to serve as prior information for regularization (step 11). Finally, the residual variables V_L_ and V_S_ are initialized to zero (step 12).

Following initialization, regularized iterative reconstruction of the hybrid CT data is performed using the split Bregman method (steps 13–18). Each global Bregman iteration repeats the following steps: (1) serial regularization with RSKR (steps 13–14) and dictionary-based sparse coding (OMP, step 15); (2) regularization residual updates (steps 16–17); and (3) data fidelity updates relative to the regularized estimates of X_L_ and X_S_, D_L_ and D_S_, at each energy (step 18). Fewer than six global Bregman iterations were required to achieve convergence (negligible change in X_L_ and X_S_ between iterations) for both the simulation and *in vivo* experiments. Convergence is achieved in such a small number of iterations given the initialization of X_L_ and X_S_ prior to regularized reconstruction and due to the data-adaptive nature of the regularization strategies employed. Following convergence of the hybrid reconstruction algorithm, material decomposition is performed to obtain maps of the relevant basis materials, C (step 19).

### Dual-source, hybrid micro-CT scanner

In this section we provide technical details regarding the setup of our dual-source, hybrid micro-CT scanner. We also provide details on several supporting algorithms required for calibration and preprocessing prior to hybrid reconstruction.

#### System configuration, data acquisition, and projection preprocessing

The EID chain of our hybrid micro-CT scanner consists of an Epsilon high frequency x-ray generator (EMD Technologies, Saint-Eustache, QC), a G297 x-ray tube (Varian Medical Systems, Palo Alto, CA; fs = 0.3/0.8 mm; tungsten rotating anode; filtration: 0.7 mm Al, 3 mm PMMA), and a XDI-VHR CCD x-ray detector (Photonic Science Limited, Robertsbridge, UK; 22 μm^2^ pixels) with a Gd_2_O_2_S scintillator. The 22 μm^2^ EID pixels were binned to 88 μm^2^ prior to image reconstruction (1002x667 88 μm^2^ pixels / projection). 360 EID projections were acquired over a single 360° rotation (1 angular increment). The source-detector and source-object distances were 80 cm and 70 cm, respectively, resulting in a geometric magnification of 1.14 times. Each projection was acquired with an 80 kVp tungsten spectrum with a 100-mA current and a 10 ms projection integration time (resulting in ~5e4 photons / line integral). The absorbed radiation dose associated with the EID scan was ~48 mGy.

The PCD chain of the hybrid micro-CT scanner consisted of a PXS-10 65W micro-focus x-ray source (Thermo Fisher Scientific, Waltham, MA; tungsten anode; filtration: 0.25 mm beryllium) and a PILATUS3 CdTe 300K PCD with a single hardware-based energy threshold (on loan from Dectris AG, Baden-Dättwil, Switzerland; 1 mm CdTe thickness; 487x619 172 μm^2^ pixels / projection) [51–55]. With this PCD chain, 400 projections were acquired over a single 360° rotation (0.9° angular increment) for each of five threshold settings: 26, 34, 37, 39, and 45 keV. The source-detector distance was 27 cm and source-object distance was 20 cm, resulting in a geometric magnification of 1.35 times. Each projection was acquired with an 80 kVp tungsten spectrum, a 251 μA current, and a 20 ms projection integration time (resulting in ~4.2e3 photons / line integral prior to considering the energy threshold). The absorbed radiation dose per PCD scan (per threshold) was ~14 mGy.

For both the simulation experiment and the *in vivo* experiment, the EID data was reconstructed with a voxel size of 88 μm^3^. The PCD data was reconstructed with a voxel size of 127 μm^3^. At these voxel sizes and magnifications, the FWHM of the point spread function, estimated from edge-spread measurements in FBP reconstructions, was approximately twice the voxel width. With respect to the Gaussian kernels used to approximate the point spread function in the algebraic forward model (*Hybrid data fidelity* sub-section), this yielded the following parameter values: FWHM_PCD_ = 0.254 mm; FWHM_EID_ = 0.176 mm; σ_r_ = 0.078 mm.

**Fig 4:**
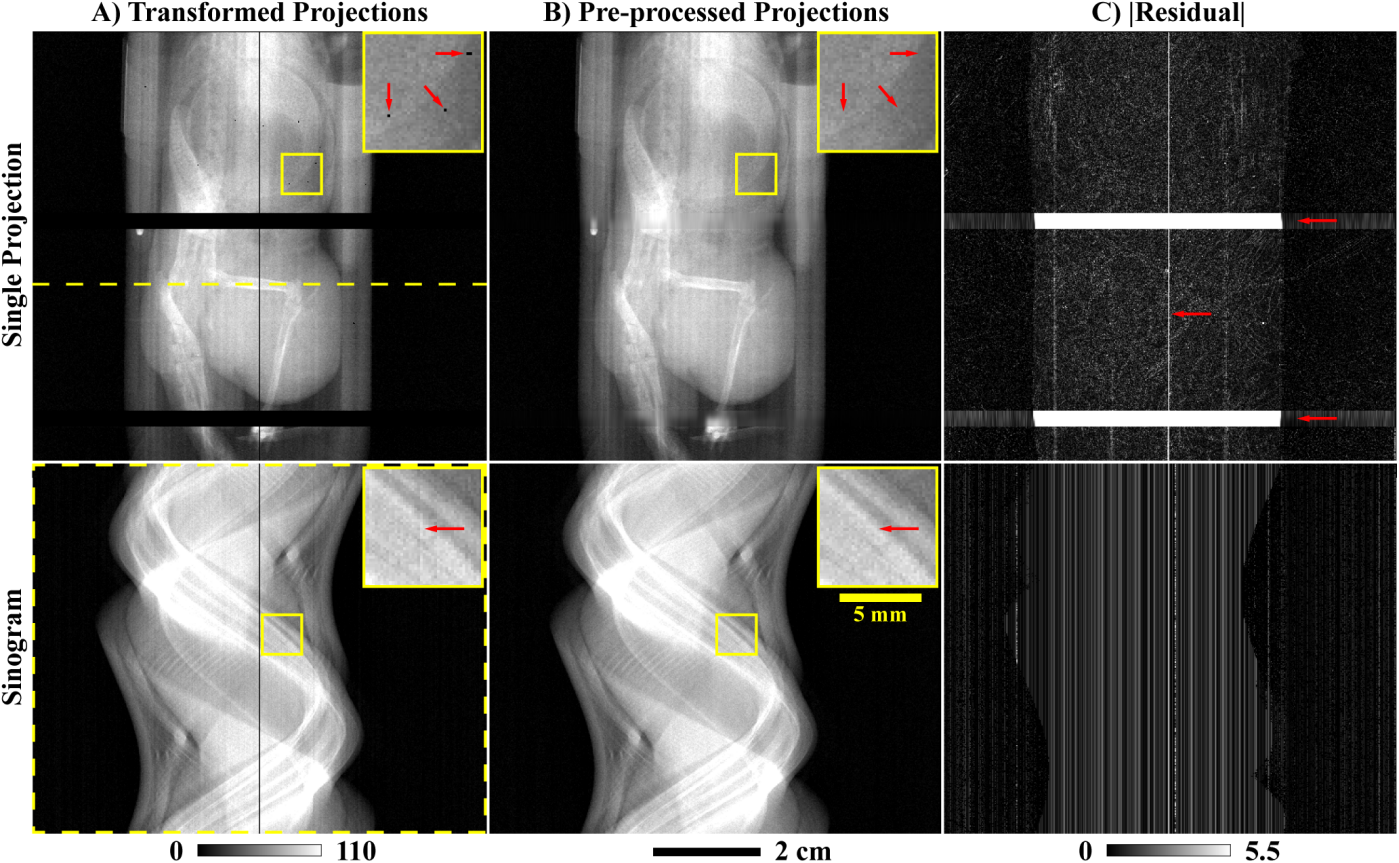
Preprocessing applied to the PCD projection data. (A) Example log-transformed PCD projection prior to processing (threshold: 26 keV). The yellow, dotted line denotes a single detector row. The readout of this row is shown as a function of angle in the bottom row of this figure (sinogram). (B) Corresponding PCD projection following the three forms of correction described in the text. In the single projection (row 1), an inset (corresponding yellow boxes) and red arrows highlight overly dark pixels before (A) and after (B) ring artifact prevention. In the sinogram (row 2), similar insets and arrows denote detector pixel readouts notably affected by ring artifact correction. (C) Absolute difference computed between (A) and (B). Note bright bands where the detector gaps were interpolated (red arrows). Also note the differences in windowing between columns (A) and (B) (below (A)) and column (C) (below (C)).

For the *in vivo* experiment and only for the PCD projection data, three preprocessing steps were performed, in order, prior to reconstruction and following bright-field normalization and log-transformation (Fig 4): (1) ring artifact prevention, (2) ring artifact correction, and (3) detector gap interpolation. (1) To prevent significant ring artifacts, permanently under- and over-exposed detector pixels were identified in exposure-averaged, bright-field images as detector pixels with signal values which deviated by more than 25% from the median signal recorded in air. Within the log-transformed projection data and at each angle, the new intensity values assigned to these outlier pixels were interpolated using a 2D Gaussian kernel (standard deviation: 1 pixel). (2) More subtle ring artifacts were corrected by estimating the intensity bias associated with specific detector pixels. Specifically, low-pass filtration of the log-transformed projection data was applied independently along the projection columns and along the angular dimension (1D box filter; length: 5 voxels or 5, 0.9° angular increments). For each detector pixel across all angles, the average difference between these two low-pass filtered sets of projections was used as a correction factor to correct the estimated intensity bias in the unfiltered projection data. The magnitude of the applied correction was not allowed to exceed the uncorrected magnitude recorded for each individual detector pixel at each projection angle. (3) As a final preprocessing step, gaps in the detector were filled in via 1D interpolation perpendicular to the orientation of the gaps. Specifically, these values were filled in using a 1D Gaussian kernel with a diameter of 41 pixels and a standard deviation of 8 pixels, with appropriate magnitude normalization. The primary vertically oriented gap was interpolated to prevent a ring artifact near the center of rotation. The larger horizontal gaps were interpolated to mitigate the deleterious impact of reprojecting partial rays during iterative reconstruction.

#### Geometric calibration and affine registration

While not exclusive to hybrid spectral CT, two related calibration operations were fundamental to the success of our *in vivo* experiment: geometric calibration and dual-chain, affine registration. The objective of geometric calibration is to determine the parameters of the system projection matrix which describe the view-dependent relationship between the x-ray source and detector positions [56]. Once determined, these parameters are used to perform projection and backprojection operations in a highly parallel fashion (e.g. on the GPU) by computing elements of the projection matrix as needed. In past work with dual chain micro-CT (i.e. with two independent sets of sources and detectors; [57]), we have applied geometric calibration to each imaging chain independently. Given well-calibrated reconstructions from each imaging chain, data from both chains can be combined in the image domain by computing an affine registration matrix. This matrix maps voxels from the secondary imaging chain into the space of the primary imaging chain. Resolution-losses due to interpolation associated with this affine transform can be mitigated by incorporating the inverse of the affine transform directly into the system projection matrix for the secondary imaging chain.

Specific to the problem of hybrid spectral CT, the geometric calibration and affine registration operations are complicated by fundamental differences between the EID and PCD data: noise level, spatial resolution, geometric magnification, field-of-view coverage, etc. Considering these differences, the precision with which the PCD data is calibrated and registered relative to the EID data will directly limit the potential for resolution enhancement through hybrid reconstruction. To achieve high-fidelity geometric calibration and registration between the EID and PCD imaging chains, we first performed geometric calibration for each chain independently at the native resolution of each imaging chain (EID: 88 μm^2^ pixels, 88 μm^3^ voxels; PCD: 172 μm^2^ pixels, 127 μm^3^ voxels). Then, using the previously described upsampling operator, B^T^, the projection matrix for the EID data, R, and the FBP operator for the PCD data, $A_{f}^{T}$, we refined the geometric calibration and affine registration of the PCD imaging chain using the EID projection data, **w**:

$$MI(w,RB^{T}A_{f}^{T}y_{avg}).$$ (56)

The fit between the two sets of data was evaluated with the mutual information similarity metric (MI, [58]). The geometric parameters for the PCD data as well as the affine transform parameters to register the PCD data into the space of the EID data (six free parameters describing translation and rotation) were directly incorporated into the PCD backprojection matrix, A^T^ ($A_{f}^{T}$). To reduce noise, the energy dimension of the PCD projection data was reduced with a variance-weighted average (variance measured in air). This resulting energy-averaged projection data, **y**_avg_, was used solely for calibration and registration. Similar to the approach in [59], geometric and affine parameters, which maximized the MI between the PCD and EID projections, were found using the covariance matrix adaptation evolution strategy (CMA-ES, [60]). Practically, the MI similarity metric appeared to be very robust to the interpolated gaps in the projection data and to the lower resolution of the reprojected PCD data.

### Digital simulation experiment

The objective of our digital simulation experiment was to establish upper bounds on the performance of the proposed hybrid spectral CT reconstruction algorithm, free from potential complications such as imperfect geometric calibration and spatial registration. The simulation experiment also allowed quantitative comparison with the expected reconstruction results for both the EID and PCD data.

#### Contrast and resolution phantom

**Fig 5:**
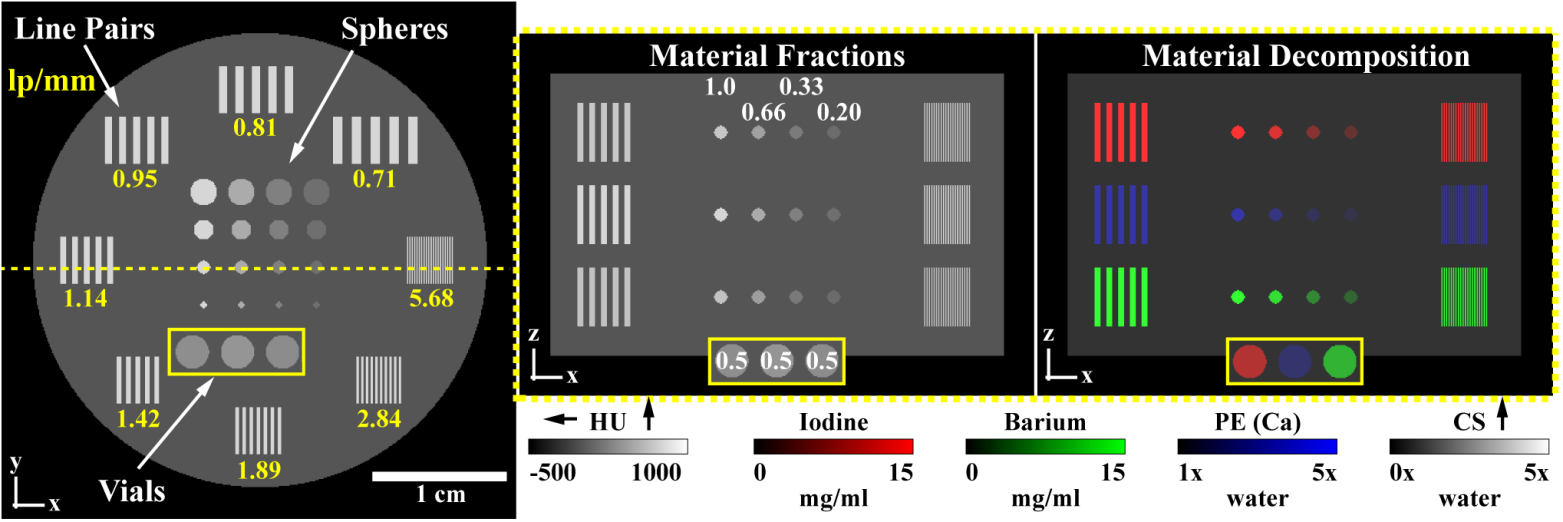
3D digital simulation phantom. The phantom we constructed for assessing our proposed hybrid spectral CT reconstruction algorithm consists of three key features: line pairs to assess spatial resolution, spheres to assess detection, and vials to make volumetric spectral measurements. These features are arranged in three disks along the z-axis. The line pairs and spheres in each disk exclusively contain one of the three contrast materials: iodine (red), calcium (blue), and barium (green). The phantom is synthesized from material fraction maps which denote fractions of the maximum concentration of each contrast material (1.0: 15 mg/ml, iodine; 15 mg/ml, barium; 75 mg/ml calcium). Here, and elsewhere in this paper, material decompositions are shown as overlaid material maps, coded by basis function (color) and concentration (intensity; multiple relative to water). The window width and level for the CT data and for each material map are as shown.

Fig 5 details the digital contrast and resolution phantom we constructed to assess the fidelity of hybrid reconstruction. The digital phantom is a variation on the ACR 464 phantom used for quality assurance in clinical CT scanners [61]. Specifically, the phantom consists of a cylinder of water with a diameter scaled to match the diameter of the cradle we use for scanning adult mice, 3.4 cm. The cylinder is divided into three segments (disks) along the z-axis. Each disk is dominated by a single contrast material we aim to separate *in vivo*: iodine (red), calcium (blue), and barium (green). The materials are present in realistic concentrations for small animal micro-CT—15 mg/ml of iodine, 75 mg/ml of calcium, and 15 mg/ml of barium in water (material fraction = 1.0). To assess spatial resolution, each disk contains a set of line pairs which discretely represent spatial frequencies from 0.71 to 5.68 line pairs per mm (lp/mm). To assess the trade-offs in feature detection with feature size and material concentration, a grid of spheres is included within each disk. Along the y-axis, the diameters of these spheres vary from 2.0 mm to 0.5 mm in increments of 0.5 mm, with some truncation due to discretization. Along the x-axis, the concentrations of each disk’s material take on the following fractions of the maximum concentration: 1.0, 0.66, 0.33, and 0.20. To monitor spectral fidelity during iterative reconstruction, volumetric measures are taken in cylindrical “vials” which contain 0.5 times the maximum concentration of each material in all three disks along the z-axis. An additional vial of water, also used for monitoring spectral fidelity, is positioned to the right of the vials visible in Fig 5.

Using this digital phantom and the previously defined scanning configurations (*System configuration* sub-section), projection measurements, I, were synthesized with the following spectral model:

$$I=\int_{e}I_{D}(e)\;\exp\left[-\int_{r}\mu (r,e)\;dr\right]\;de,$$ (57)

$$\mu (r,e)=\sum_{m=1}^{n_{m}}\left[\frac{\mu }{\rho }(m,e)*c_{max}(m)*frac(m,r)\right].$$ (58)

Specifically, given the maximum concentration, c_max_(*m*), for each material, *m* (n_m_ = 4 basis materials), and the material fraction maps used to construct the phantom, frac(*m*,*r*) (Fig 5), the linear attenuation coefficients (Eq 58) were integrated as a function of position, *r*, and energy, *e* (Eq 57). The mass attenuation coefficients, $\frac{\mu }{\rho }(m,e)$, were derived from *Spektr* [62]. For the EID projection data, the detected signal in the absence of the phantom, I_D_(*e*), was modeled as follows:

$$I_{D}(e)=I_{0,EID}(e)*S_{EID}(e)*e.$$ (59)

I_0,EID_(*e*) denotes the 80 kVp source spectrum previously described in the *System configuration* sub-section. S_EID_(*e*) denotes the normalized detector sensitivity function, which includes the response of the Gd_2_O_2_S scintillator and additional PMMA filtration. For the PCD projection data, the detected signal model included a single hardware-based energy threshold, *t*, consistent with the specifications of the PILATUS3 detector:

$$I_{D}(e)=I_{0,PCD}(e)*S_{PCD}(e)*\int_{g}\left[{rect}_{t}(e-g)*G_{esf}(g,\sigma_{esf})\right],$$ (60)

$${rect}_{t}(e)=\left\{ \begin{array}{c} 0,\;e<t\\ 1,\;e\geq t \end{array}\right..$$ (61)

**Fig 6:**
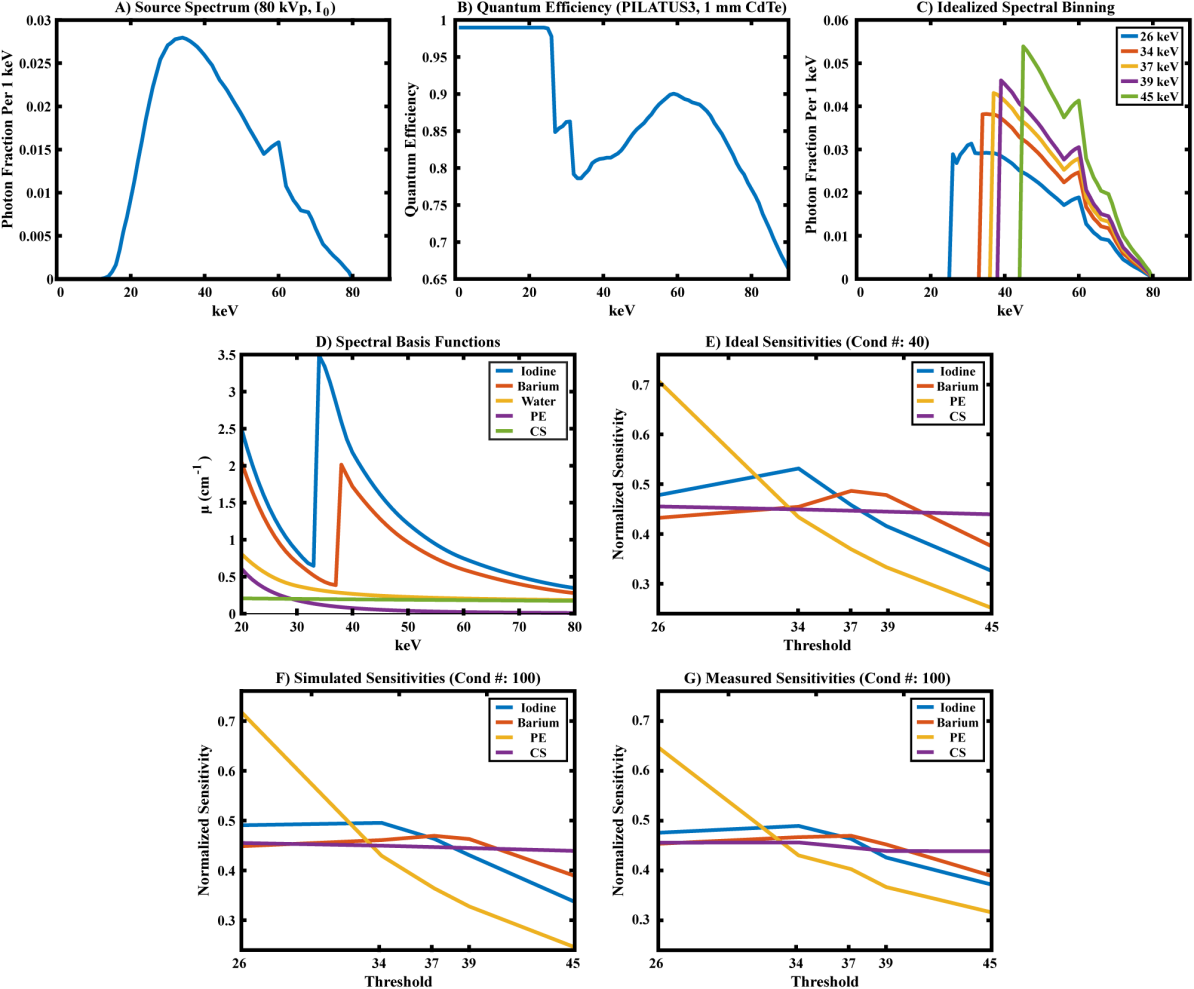
Spectral modeling of PCD data acquisition. (A) 80 kVp tungsten source spectrum. (B) Quantum efficiency for 1 mm of CdTe in the PILATUS3 detector. (C) Idealized spectral binning (rect functions) applied to the element-wise product of the source spectrum and the detector sensitivity function for each of the energy thresholds used for imaging in this paper. (D) Spectral basis functions considered in this work. Note that the PE and CS basis functions are scaled to sum to the attenuation of water (each with a coefficient of one). The attenuation coefficient curves for iodine and barium are scaled to a density of 20 mg/ml for display purposes. (E) Unit-norm material sensitivity vectors for each of the basis materials, as predicted by the spectral model. (F) Unit-norm material sensitivity vectors as predicted by the spectral model, including a Gaussian energy-spread function with a standard deviation of 3.25 keV, to match the conditioning of sensitivity vectors derived for our *in vivo* experiment (G).

Again, the 80 kVp source spectrum, I_0,PCD_(*e*), was as described in the *System configuration* sub-section. The normalized PCD sensitivity function, S_PCD_(*e*), included the expected quantum efficiency of detection with 1 mm of CdTe (Fig 6B; [63]), but did not model more complex physical phenomena such as charge sharing and pulse pile-up. Rectangular (rect) functions (Eq 61) were used to represent idealized spectral bins. The components of the PCD spectral model are summarized in Fig 6.

Given these basic spectral models for the EID and PCD data acquisition, additional steps were taken to better match the (1) image noise, (2) spatial resolution, and (3) energy resolution characteristics of the *in vivo* data (*In vivo experiment* sub-section). To reproduce the observed noise levels (1), the total number of counts associated with I_0,EID_ (5e4 photons / line integral) and I_0,PCD_ (4.2e3 photons / line integral) were empirically determined. Specifically, these values were adjusted such that when the recorded counts, I, (Eq 57) were drawn from a Poisson distribution with a mean equal to I, the resultant FBP reconstructions reproduced the expected noise standard deviations measured in water. For the EID data, the noise standard deviation in water was ~80 HU (88 μm^3^ voxels). For the PCD data, the noise standard deviations ranged from ~200 HU (26 keV threshold) to ~450 HU (45 keV threshold; 127 μm^3^ voxels). Prior to the computation of the projection data and to approximately reproduce the observed spatial resolution (2), the material density maps (c_max_(*m*) * frac(*m*,*r*), Eq 58), discretized with 88 μm^3^ voxels, were resampled with the B operator. As previously reported, the Gaussian FWHM for the approximated point spread function of the EID data was 176 μm, while the FWHM for the PCD data was 254 μm. The B operator applied to the PCD data also resampled the voxel size from 88 μm^3^ to 127 μm^3^.

Finally, to better model the limited energy resolution of the PCD (3), the rect function used for ideal spectral binning (Eq 61) was convolved with an energy-invariant, Gaussian energy spread function, G_esf_ (Eq 60), parameterized by *g*, the energy offset from *e*, and σ_esf_, the standard deviation of the energy spread function. The energy (*e*) dependence of the energy spread function for CdTe-based PCDs is well documented [16]; however, in the absence of a precise model for this dependence in the PILATUS3 detector, we used an alternative approach to make the simulation more realistic. Specifically, we chose a fixed value for the σ_esf_ parameter such that the resultant material sensitivity matrix derived from the simulated data had a condition number closely matching that of the material sensitivity matrix derived from the *in vivo* data. Mathematically, the condition number is computed as the ratio of the largest and smallest singular values of a matrix, and it quantifies the potential for error amplification in solving linear inverse problems (e.g. material decomposition). Fig 6E plots the simulated material sensitivities, following magnitude normalization per material and using ideal (rectangular) spectral bins (condition number = 40). By contrast, Fig 6F plots the same simulated, normalized material sensitivities when an energy-invariant Gaussian energy spread function is included in the forward model (σ_esf_ = 3.25 keV; condition number = 100). While not identical to the material sensitivities derived from the real data (Fig 6G), the degradation of iodine (34 keV threshold) and barium (37, 39 keV thresholds) K-edge contrast is convincingly reproduced.

Note that the phantom is constructed from calcium, water, barium, and iodine, but the phantom is decomposed into basis functions for the PE, CS, barium, and iodine. Our PE and CS basis function were derived using the analytical, energy-dependent expressions for the PE and CS [46] and energy-effective attenuation calculations using our forward model (Eq 57). These derived PE and CS basis functions were then modified to exactly describe the derived basis functions for calcium and water across all five PCD thresholds and the EID data. Specifically, we projected the derived PE and CS basis functions onto the orthonormal subspace defined by the basis functions for calcium and water (<3% difference before and after projection). This fitting procedure was important to maximize the separation of the PE and CS basis functions from the basis functions for iodine and barium (i.e. to minimize the condition number of the material sensitivity matrix). For convenience, the PE and CS basis functions were also scaled to each decompose to a coefficient of one in pure water. Setting the lower bound of the PE display window to one then visually differentiated calcium from water in overlaid material decompositions (Fig 5).

#### Quantitative evaluation

Several metrics were used to quantitatively evaluate the results of hybrid spectral CT reconstruction using the digital phantom. To globally assess the fidelity of the reconstructed results at a single energy, **x**_*e*_, we computed the root-mean-square error (RMSE) relative to the expected reconstruction results, ${\bar{x}}_{e}$:

$$RMSE(x_{e},{\bar{x}}_{e})=\sqrt{\frac{1}{n_{x}}\sum_{i=1}^{n_{x}}{\left(x_{e,i}-{\bar{x}}_{e,i}\right)}^{2}}.$$ (62)

Here, *i* indexes all n_x_ voxels of the reconstructed volume at a single energy. To assess spectral fidelity independent of denoising performance, we also computed the absolute bias within volumetric regions of interest expected to have constant intensity, k:

$$bias(x,k)=\left|\frac{1}{n_{roi}}\sum_{r}\left(x(r)-k\right)\right|.$$ (63)

The spatial vector **r** indexes the n_roi_ total voxels within the defined volumetric region of interest. To assess spatial resolution in the reconstructed results, we computed the modulation transfer (MT) at each spatial frequency sampled by the bar patterns in the digital phantom:

$$MT(a,b)=\frac{\left|a-b\right|}{a+b}.$$ (64)

The variables a and b correspond with average attenuation measurements taken from the expected locations of a set of contrast-enhanced lines and from the corresponding, expected gaps between the lines, respectively. Prior to the MT calculation, the expected attenuation of water was subtracted from each measurement. Any negative values of MT were set to zero. Given a vector of 120 independent MT measurements, **n**, taken in the digital phantom (n_*l*_ = 120; 8 sets of line pairs, 3 disks, 5 thresholds; Fig 5), a Gaussian modulation transfer function (MTF) was fitted to match the observed measurements under a least-squares penalty:

$$MTF(l_{i},\sigma )=\exp\left(-\frac{{l_{i}}^{2}}{2\sigma^{2}}\right),$$ (65)

$$\sigma =\begin{array}{c} \arg min\\ \sigma \end{array}\sum_{i=1}^{n_{l}}{\left(MTF(l_{i},\sigma )-n_{i}\right)}^{2}.$$ (66)

The standard deviation of the MTF, σ, is the only free parameter. The vector **l** contains the spatial frequencies in lp/mm for each corresponding measurement.

Finally, the detectability index was computed to assess the visibility of the spherical lesions within the digital phantom under a non-prewhitening observer model [64]:

$$\text{Detectability}=\sqrt{\frac{{\left(\sum_{j}{\left({MTF}_{3\text{D}}(j,\sigma )W(j)\right)}^{2}\right)}^{2}}{\sum_{j}NPS(j){\left({MTF}_{3\text{D}}(j,\sigma )W(j)\right)}^{2}}}.$$ (67)

Specifically, the detectability index was computed over a cubic region of interest defined around each spherical lesion (side length: 32 voxels; no overlap between regions). All voxels within each cubic region were indexed by the Cartesian offset vector, **j**. Specific to our implementation of the detectability index calculation, MTF_3D_ denotes the 1D MTF from Eq 65 extrapolated to 3D in an isotropic fashion based on the Euclidean distance of each voxel center from the origin of each region of interest. We defined the task function, W, as the discrete Fourier transform of the same cubic region of interest within the expected reconstruction following the subtraction of the attenuation of water from each voxel. The local noise power spectrum, NPS, was approximated as the power spectral density of the residuals computed between the hybrid reconstruction results and the expected reconstruction results within each cubic region of interest.

**Fig 7:**
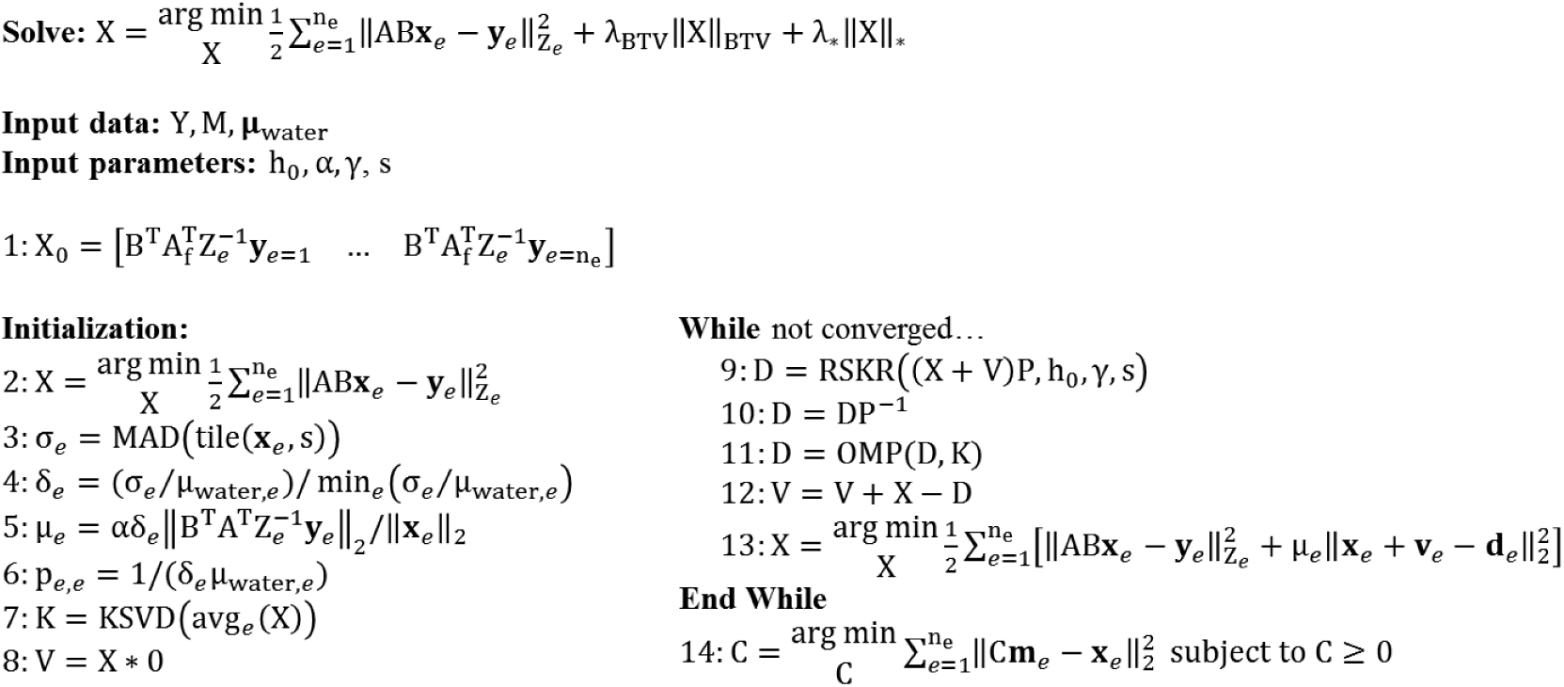
Pseudocode for PCD only reconstruction with the split Bregman method. As a control experiment for the proposed hybrid spectral CT reconstruction algorithm (Fig 1), which uses both EID (**w**) and PCD (Y) projection data, we performed a second iterative reconstruction using only the PCD projection data. This PCD only reconstruction algorithm is largely analogous to the hybrid reconstruction algorithm, with two main exceptions. First, the PCD data is reconstructed directly (X) rather than indirectly as a function of the EID reconstruction (X_L_) and the spectral contrast provided by the PCD data (X_S_). Second, during initialization, the dictionary is trained using a variance ($\sigma_{e}^{2}$) weighted average of the initialized reconstruction results (avg_*e*_(X), step 7) rather than using a FBP reconstruction of the EID data (Fig 1, step 11).

Our primary use of the detectability index is to compare the results of hybrid spectral CT reconstruction with the results of a second control reconstruction where the EID projection data is not used. We refer to the results of this control experiment as “PCD only” results. The algorithm applied for PCD only reconstruction is very similar to the algorithm applied for hybrid reconstruction, which includes the EID data (compare the pseudocode in Fig 7 with Fig 1). However, there are two significant differences. First, the PCD data is reconstructed directly (X) rather than indirectly as a function of the EID reconstruction (X_L_) and the spectral contrast provided by the PCD data (X_S_). Second, during initialization, the dictionary is trained using a variance ($\sigma_{e}^{2}$) weighted average of the initialized reconstruction results (avg_*e*_(X), step 7) rather than using a FBP reconstruction of the EID data (Fig 1, step 11). Given these changes, the purpose of the PCD only control experiment is to establish a base-line for the resolution enhancement and denoising performance improvements afforded by the incorporation of EID data into the PCD reconstruction problem.

### *In vivo* experiment

To validate the proposed hybrid spectral CT reconstruction algorithm and to justify several simplifying assumptions made in its derivation (e.g. spatially-invariant Gaussian point spread function, log-transformation of energy-dependent line integrals, etc.), we acquired *in vivo* mouse data using the hybrid micro-CT system and data acquisition parameters described in *System configuration* sub-section. Notably, the image reconstruction and regularization code used to reconstruct the simulated data was identical to the code used to reconstruct the *in vivo* data, for both hybrid spectral CT reconstruction and for PCD only reconstruction. The *in vivo* experiment was conducted in an adult mouse model of soft-tissue sarcoma (*LSL-Kras*^G12D/+^*Trp53*^FL/FL^ conditional mutants) with sarcoma tumor growth initiated by intramuscular injection of adenovirus expressing Cre recombinase (see [65]). Three days prior to scanning, the mouse was injected with the barium-based nanoparticle contrast agent ExiTron nano 12000 (Miltenyi Biotec, Bergisch Gladbach, Germany) at a dose of 0.15 ml per 25 g mouse. Immediately prior to scanning, the same mouse was injected with blood pool, liposomal iodine contrast agent [66] at a dose of 0.3 ml per 25 g mouse. Following the paradigm we established in previous work [6], the purpose of delayed-phase imaging was to the allow the barium contrast to accumulate within the sarcoma tumor based on the enhanced permeability and retention effect [67]. Given three days for the ExiTron nano to clear from the vasculature, iodine liposomes were then injected to allow differentiation of tumor vasculature from extravasated barium via spectral CT and material decomposition.

Referring back to the material sensitivity fitting procedure performed in the digital phantom (*Contrast and resolution phantom* sub-section), a similar procedure was used to derive spectral basis functions for the *in vivo* data (Fig 6G), as a prerequisite for material decomposition. Specifically, the model-derived PE and CS basis functions were projected onto a subspace defined by attenuation measurements taken in water, polylactic acid (mouse cradle material), and cortical bone in the EID and PCD data. The material subspace was defined by performing a singular value decomposition on the attenuation measurements following unit normalization of each material sensitivity vector. Given two degrees of freedom (PE, CS), only the first two singular vectors were used to define the subspace (<4% difference before and after projection). For robustness to measurement variability, the rank two approximation for the attenuation of water was subtracted from the aqueous iodine and barium attenuation measurements to derive spectral basis functions for iodine and barium. The iodine and barium attenuation measurements were taken in separate calibration vials included within the field-of-view of the *in vivo* scans (10 mg/ml iodine, 6.5 mg/ml barium).

### Ethics statement

The *in vivo* mouse experiment in this work was conducted in accordance with the governing protocol approved by the Institutional Animal Care and Use Committee of Duke University Medical Center (protocol A251-14-10). Specifically, the sarcoma tumor mouse was bred in the laboratory of our collaborator, Dr. David Kirsch (Duke University Medical Center, Radiation Oncology). The animal was housed in an American Association for Assessment and Accreditation of Laboratory Animal Care (AAALAC) approved barrier facility managed by Duke’s Division of Laboratory Resources. Sarcoma tumor growth was initiated by intramuscular injection of adenovirus expressing Cre recombinase at approximately one month of age. Micro-CT scanning was performed approximately three months after injection. During micro-CT scanning, the mouse was free breathing under anesthesia using 2±0.5% isoflurane delivered by a nose cone. Following scanning, the mouse was sacrificed by anesthetic overdose (250 mg/kg pentobarbital sodium; Euthasol from Virbac AH, Inc. of Fort Worth, TX), followed by thoracotomy.

The Duke University Medical Center animal management program is accredited by the AAALAC and meets National Institutes of Health standards as set forth in the “Guide for the Care and Use of Laboratory Animals” (NIH Publication No. 85–23). Duke University Medical Center also mandates compliance with the ‘‘Public Health Service Policy on Humane Care and Use of Laboratory Animals by Awardee Institutions” and the NIH “Principles for the Utilization and Care of Vertebrate Animals Used in Testing, Research and Training.”

## Results

In this section we summarize the results of applying the proposed hybrid CT reconstruction algorithm to simulated and *in vivo* spectral micro-CT data. We also summarize the computational resources and time required to execute the proposed algorithm.

### Simulations

**Fig 8:**
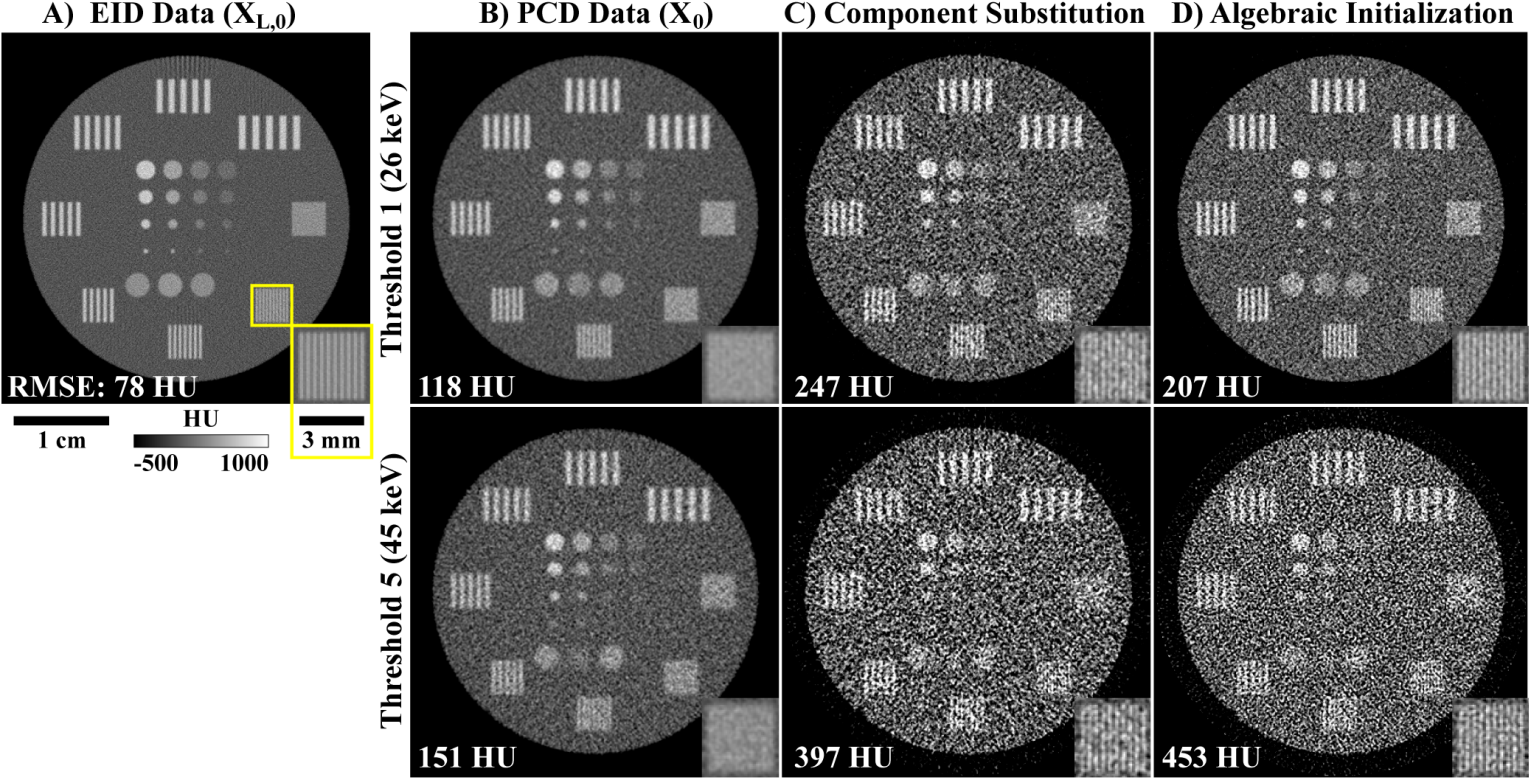
Simulated results of component substitution and algebraic initialization in hybrid reconstruction. (A) Initial FBP reconstruction using the EID projection data. A magnified inset (yellow box) shows the set of line pairs with a spatial frequency of 2.84 lp/mm. Note that the magnified inset (only) is intensity averaged over 21 consecutive, 2D slices for improved visibility. (B) Initial upsampled, FBP reconstructions using the PCD projection data corresponding with thresholds of 26 (lowest noise level) and 45 (highest noise level) keV by row (Fig 1, step 1). (C) The results of component substitution (X_L,0_ + X_S,0_; Fig 1, steps 3–4). (D) Results of algebraic initialization (Fig 1, step 5). The RMSE, computed over the entire reconstructed volume, is reported in HU at the bottom-left corner of each panel. Note that all simulation results in this paper are shown with a voxel size of 88 μm^3^.

Following the pseudocode in Fig 1, Fig 8 summarizes the component substitution and algebraic initialization steps of hybrid reconstruction, which are performed prior to regularized, iterative reconstruction. Specifically, Fig 8A shows a single 2D slice through the iodine disk of the FBP reconstruction of the EID data. A magnified inset (yellow box) highlights a set of line pairs (2.84 lp/mm) that is clearly resolved in the EID data, but is not resolved in the upsampled PCD reconstructions (Fig 8B). Following component substitution (Fig 8C), the objective of fusing the spatial resolution of the EID data with the spectral contrast of the PCD data is apparently achieved, as the line pairs are again visually obvious (following intensity averaging over 21 consecutive slices in the inset); however, the noise level in the reconstructed results increases substantially. This increase in noise is a consequence of the poor conditioning of the material sensitivity matrix used to synthesize and subtract a low-resolution estimate of the EID data (Fig 1, steps 2–3), motivating the need for regularized, iterative reconstruction. Prior to regularized, iterative reconstruction, the estimates for X_L_ and X_S_ are algebraically refined (Fig 1, step 5) to reconcile them with both sets of projection data under the hybrid data fidelity terms. Interestingly, this algebraic initialization step reduces the global RMSE for the reconstruction with a threshold of 26 keV, but increases it for the data with a threshold of 45 keV. This discrepancy is, presumably, explained by larger attenuation differences between the EID and PCD reconstructions when a high-energy threshold is used.

**Fig 9:**
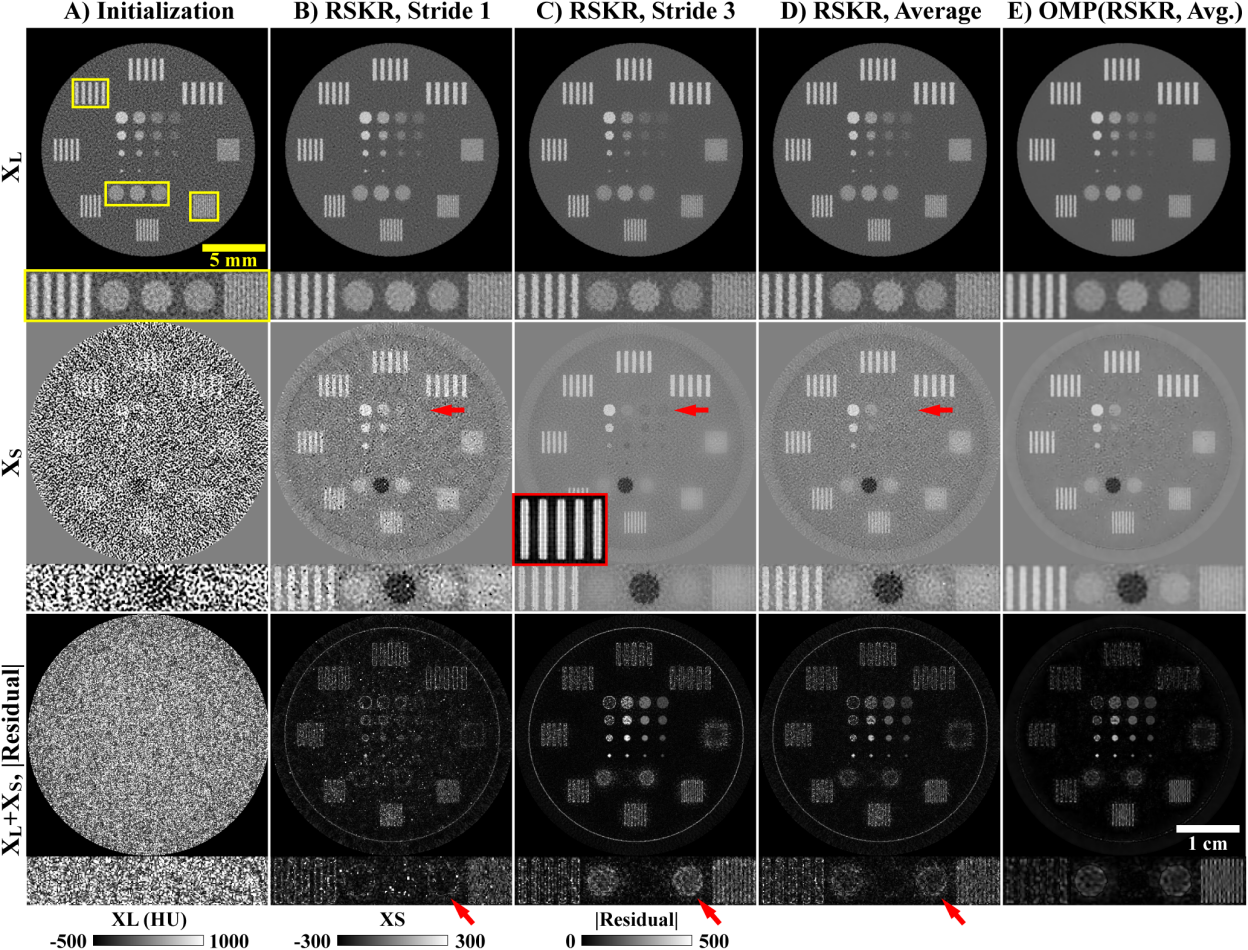
Regularization during the first iteration of hybrid reconstruction. (A) Initialization results for the 37 keV threshold in the iodine disk. Rows show X_L_ (EID reconstruction), X_S_ (PCD contrast), and the absolute difference between X_L_ + X_S_ and the expected reconstruction result (|Residual|). In the first row, yellow boxes denote three regions of interest which are concatenated and magnified at the bottom of each panel for improved visibility. Note that these regions of interest represent a single 2D slice. (B) RSKR applied to (A) without tiling (s = 1). (C) RSKR applied to (A) with tiling (s = 3). A contrast-enhanced and 21-slice averaged inset in the second row (red box) highlights striping artifacts introduced because of tiling. (D) RSKR applied to (A) and following the steps outlined in Fig 3 steps 4–8 (“Average”). Red arrows denote intensity bias introduced when striding is used (row 2: contrast inversion in X_S_; row 3: localized bias in X_L_ + X_S_). (E) OMP applied to (D).

Given a clear need for robust regularization to reduce noise while preserving the enhanced spatial resolution, Fig 9 summarizes the application of RSKR and sparse coding with OMP during the first iteration of regularized reconstruction (Fig 1, steps 13–15). In the first column, the algebraic initialization results are shown in a single 2D slice through the iodine disk of the 37 keV threshold data. To highlight the transfer of image structure from the EID data to the PCD data, X is broken down into its constituents, X_L_ and X_S_, by row. Columns (B)-(D) compare three variations of RSKR, each independently applied to the initialization (A). Specifically, (B) shows the results of applying RSKR without tiling (i.e. no stride; s = 1). Comparing X_S_ between (A) with (B), there is an obvious and dramatic improvement in the signal-to-noise ratio and the visual correspondence in image structure between X_L_ and X_S_. Looking at the absolute residual image (row 3), which is computed relative to the expected reconstruction of X, further reveals that the bias in the regularized result is negligible relative to the amount of noise removed. Comparing X_S_ between (B) with (C), illustrates the impact of applying RKSR with tiling (s = 3). Significant additional noise reduction is observed because of the higher MAD measured in the tiled data (Fig 2) and used to scale the regularization strength (R_joint_, Eq 29). Furthermore, low frequency background noise is significantly removed as a function of effectively increasing the size of the filtration domain. Unfortunately, RSKR with tiling introduces significant additional bias due to non-local intensity averaging (red arrows) and subtle artifacts associated with the phase of the tiling operations (red box in (C), row 2).

To achieve a compromise between the spectral fidelity of RSKR without tiling (Fig 9B) and the superior denoising performance of RSKR with tiling (C), we adopted a simple, yet ultimately effective, averaging scheme. Specifically, we performed both tiled and untiled joint BF (“jBF”) and then averaged the results together during each iteration of RSKR (Fig 3, steps 4–8). Visual inspection of the regularized results for X_S_ illustrate that this averaging scheme effectively suppresses the most significant outliers remaining in (B) at a lower level of bias than the results in (C) (red arrows). Outlier suppression and the attenuation of low frequency noise prove to be effective preconditioning steps for sparse coding with OMP (E), which is applied to the output of RSKR (D). Notably, while the visual differences between the three forms of RSKR applied to X_L_ are subtle due to the dominance of X_L_ in the most significant singular vector (*RSKR* sub-section), improvements in image homogeneity and in the definition of edge features are clear for both X_L_ and X_S_ following OMP. Furthermore, because the mean of each patch is explicitly preserved during OMP, no substantial increase in intensity bias is observed in the absolute residual images.

**Fig 10:**
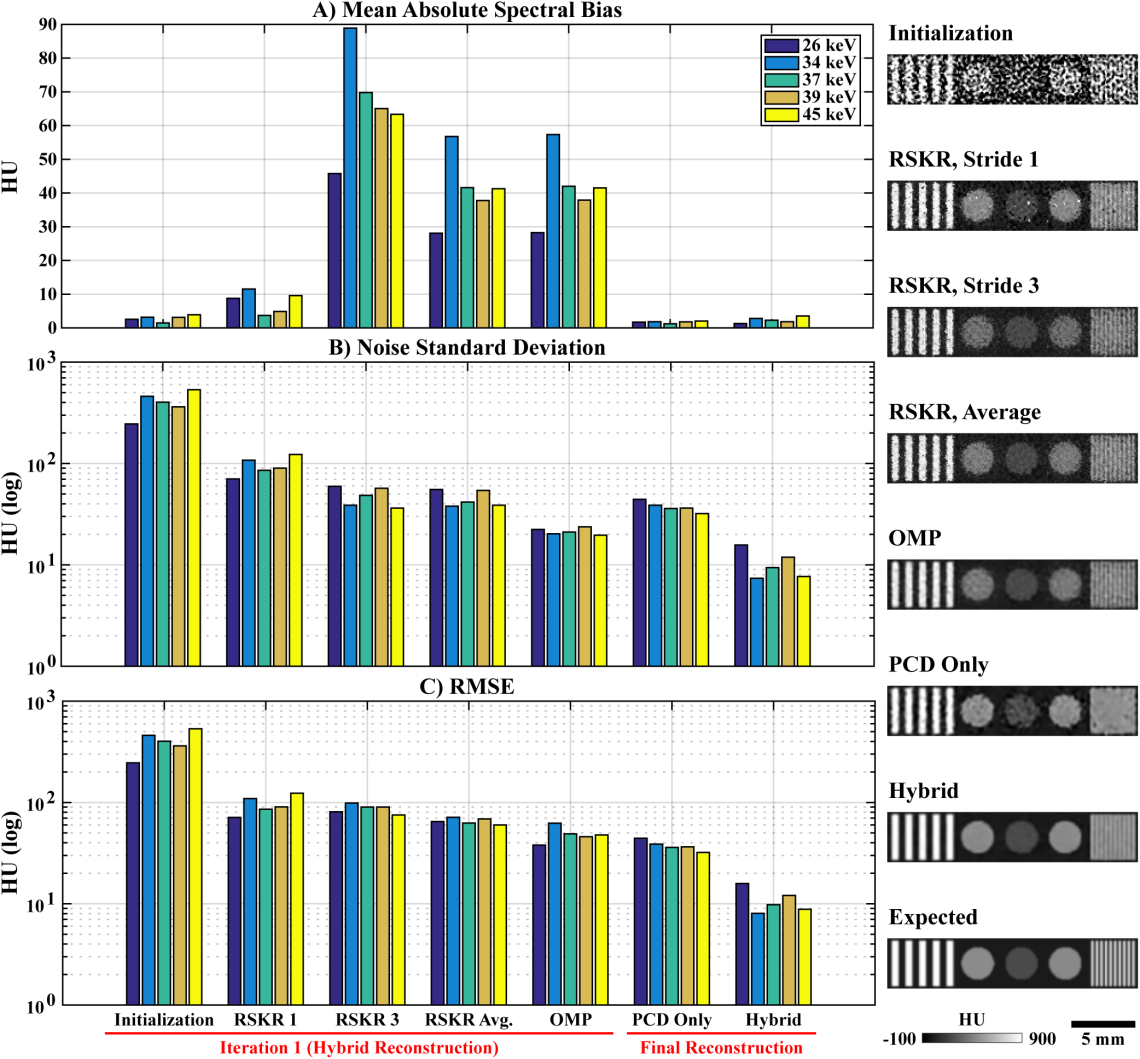
Quantitative analysis of the simulated regularization and reconstruction results. These measurements were taken in each of the phantom’s calibration vials and then averaged to yield a single value per energy threshold ((A) absolute bias, Eq 63; (B) standard deviation; (C) RMSE, Eq 62). The first five sets of bars (“Iteration 1 (Hybrid Reconstruction)”) match the experimental conditions of the results summarized in Fig 9. The last two sets of bars (“Final Reconstruction”) represent the final reconstruction results after 6 iterations of the split Bregman method (“PCD Only”: Fig 7, steps 9–13; “Hybrid”: Fig 1, steps 13–18). Borrowing from the layout in Fig 9, the bar plots are accompanied by a visual comparison of the results (right column; 37 keV threshold; X_L_ + X_S_).

The plots in Fig 10 quantitatively confirm the observations made with respect to Fig 9 and extend the analysis to all five PCD thresholds. The scalar values plotted in Fig 10 are the averages of independent, volumetric measurements taken in each of the spectral calibration vials of the digital phantom (4 material vials in each of 3 disks; Fig 5). The first 5 sets of bars (“Iteration 1”) correspond with the experimental conditions presented visually in Fig 9A–9E, comparing the algebraic initialization results with three variations on RSKR and sparse coding with OMP applied during the first iteration of hybrid reconstruction. Notably, RSKR without tiling (“RSKR 1”, s = 1) is seen to introduce a negligible level of bias relative to the algebraic initialization (Fig 10A) considering that it removes several hundred HU of noise (Fig 10B). RSKR with tiling (“RSKR 3”, s = 3) is seen to further reduce the noise standard deviation at the expense of a significant increase spectral bias. Interestingly, the proposed averaging strategy (“RSKR Avg.”) markedly reduces the resultant spectral bias with little change in the achieved denoising performance. The RMSE plot confirms this synergy ($RMSE=\sqrt{{bias}^{2}+{SD}^{2}}$; Fig 10C), reporting the lowest error for each threshold using the averaging approach. Consistent with the previous visual assessment, applying sparse coding with OMP following RSKR with averaging further reduces noise without increasing spectral bias, lower the RMSE further.

Following OMP, the regularized results are reconciled with the original projection data in the data fidelity update step (Fig 1, step 18). With respect to the plots in Fig 10, the purpose of the data fidelity update step is to strike a balance between the spectral bias introduced by regularization and the noise (rank) reduction achieved through regularization. Thanks to regularization residual tracking (Fig 1, steps 16–17) and the data-adaptive nature of our regularization scheme, proportionally less spectral bias is introduced during subsequent Bregman iterations. Using the previously described regularization parameters (*Rank-sparsity constrained* sub-section), convergence with respect to the original objective function was achieved within 6 Bregman iterations for all regularized iterative reconstructions in this work. For the digital simulation experiment, the last set of bars in Fig 10 corresponds with these “Final” “Hybrid” reconstruction results. The spectral bias introduced by regularization is virtually eliminated in the final reconstruction results, while additional gains are made in reducing the noise level and the RMSE. Impressively, the proposed iterative reconstruction scheme reduces the RMSE in the final reconstructed results by more than an order of magnitude relative to the algebraic initialization.

**Fig 11:**
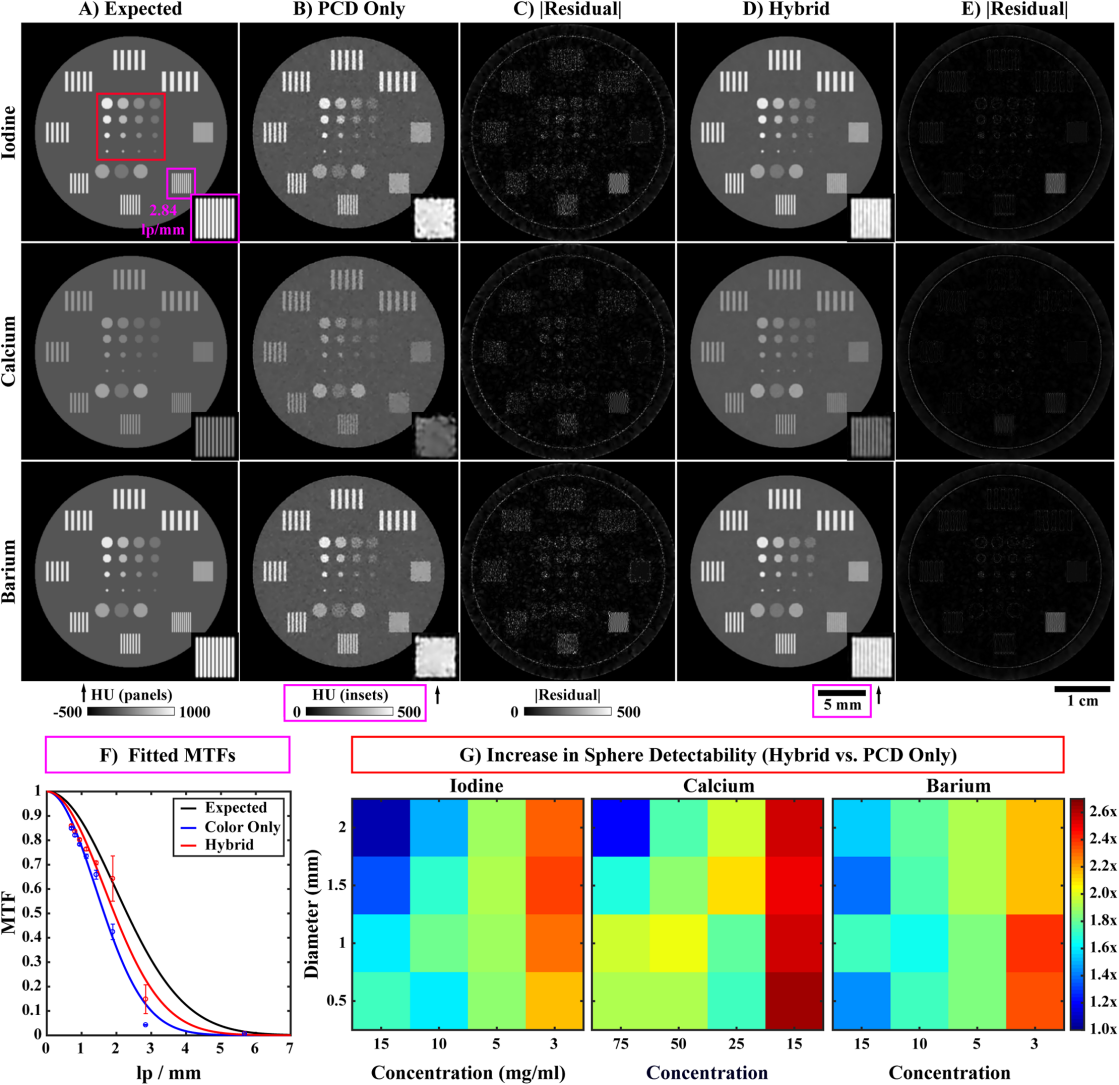
Comparison of hybrid and PCD only reconstruction results using identical PCD projection data. (A) Expected reconstruction results in a single 2D slice through the center of each material disk (37 keV threshold). The magenta box denotes a set of line pairs that are magnified and contrast-enhanced for comparison (single 2D slice; 2.84 lp/mm). The red box denotes the spherical lesions used for detectability analysis in (G). (B) Final PCD only reconstruction results (X). (C) Absolute difference between (A) and (B). (D) Final hybrid reconstruction results (X_L_ + X_S_). (E) Absolute difference between (A) and (D). (F) Gaussian MTFs fitted from MT measurements taken in all 3 material disks and all 5 energy thresholds (error bars: ±1 SD). (G) The increase in the detectability index associated with hybrid reconstruction over PCD only reconstruction for each of the spherical lesions. The results are organized by material disk, diameter, and material concentration (in mg/ml) and are averaged over all 5 energy thresholds.

The last two sets of bars in Fig 10A–10C compare the final hybrid reconstruction results (EID and PCD projection data; Fig 1) and the control reconstruction results (PCD projection data only; Fig 7). The inclusion of the higher-fidelity, and higher-resolution EID projection data in the hybrid reconstruction problem reduces the noise standard deviation and RMSE by approximately three times at each energy threshold without increasing the level of spectral bias in the final results. This result is particularly interesting considering the substantial increase in noise originally associated with component substitution, which is performed for hybrid reconstruction only (Fig 8). As visually summarized in Fig 11A–11E for the 37 keV threshold, these improvements apply uniformly over all three material disks of the phantom. Closer analysis of the set of line pairs with a spatial frequency of 2.84 lp/mm (magenta box in (A)) reveals that the resolution enhancement first introduced by component substitution (Fig 8) is at least partially preserved in the hybrid reconstruction results. The PCD only reconstruction does not resolve this set of line pairs in any of the material disks. Quantitatively, these observations are justified in Fig 11F, which compares the expected MTF derived from the Gaussian point spread function ((A), black) with fitted Gaussian MTFs derived from measurements in the hybrid (red) and PCD only reconstruction results (blue; measurements fitted over all 3 materials disks and 5 thresholds). Interestingly, the MT values measured for 1.89 and 2.84 lp/mm are more highly variable in the hybrid results than in the PCD only results (error bars: ±1 SD).

Fig 11G further compares the hybrid and PCD only results using the detectability index computed over cubic regions of interest defined around each spherical lesion (red box, (A); *Quantitative evaluation* sub-section). Specifically, panel (G) displays energy-averaged ratios of the detectability index computed for the hybrid and PCD only reconstructions as a function of material disk, lesion diameter, and material concentration. According to these plots, the hybrid reconstruction results exhibit significantly improved low-contrast detectability relative to the PCD only reconstruction results, with detectability improvements as high as 2.6x for the lowest contrast material disk (calcium). While the hybrid reconstruction results exhibit some level of detectability improvement for all of the spherical lesions, improvements in detectability as a function of decreasing feature size appear to be more modest (≤ 2.0x for the highest contrast lesions).

**Fig 12:**
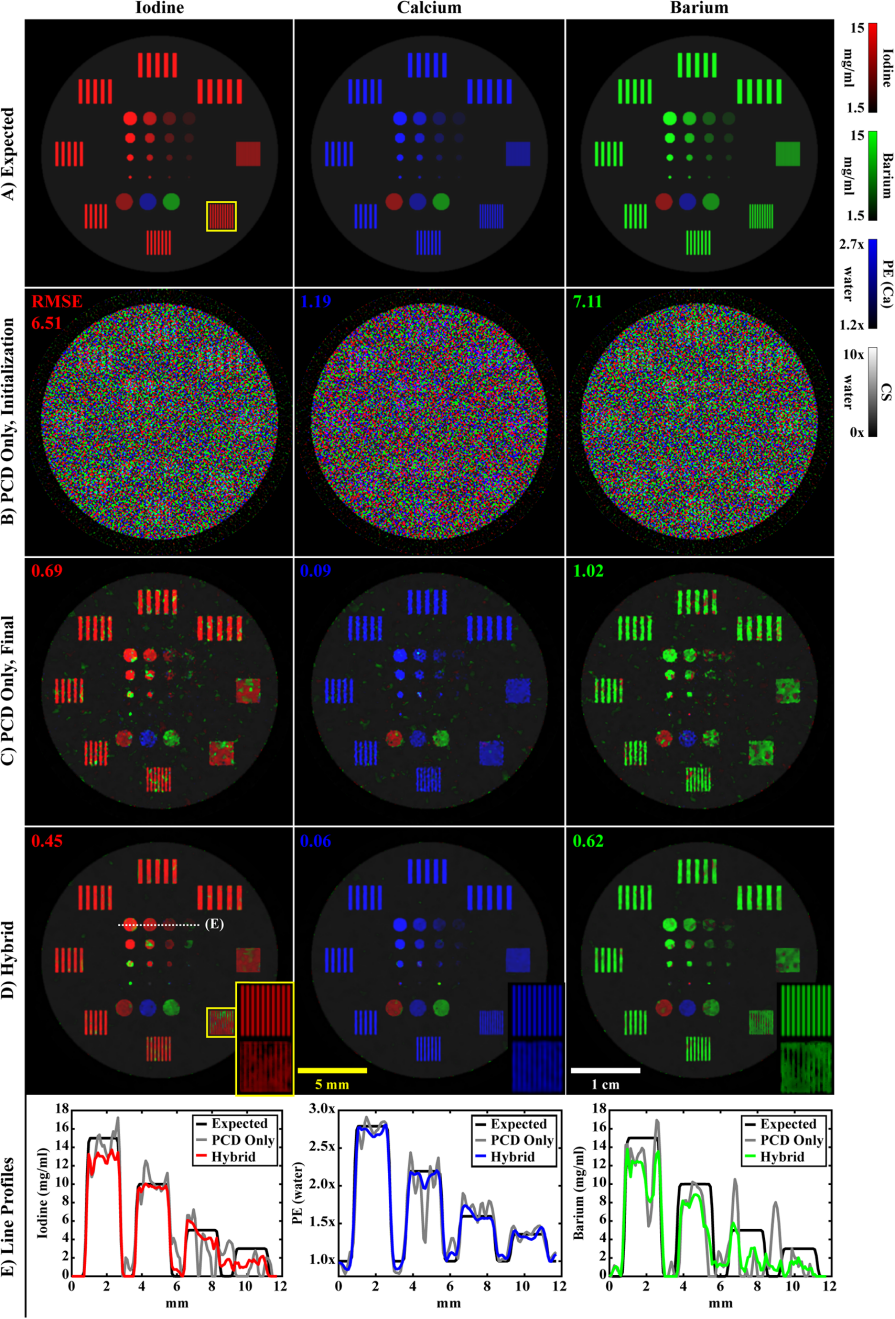
Material decomposition results for the phantom simulation experiment. (A) Expected material decomposition shown in the central 2D slice of each material disk. (B) Reference material decomposition performed following algebraic initialization (PCD only reconstruction; Fig 7, step 2). (C) Final PCD only material decomposition results (Fig 7, step 14). (D) Final hybrid material decomposition results (Fig 1, step 19). The global RMSE computed for each expected material map is shown in the upper-left of each panel in the assigned units (see calibration bars, upper-right). Insets (yellow boxes in column 1) compare the expected decomposition results (inset, top) with the final hybrid results (inset, bottom; 2.84 lp/mm; expected material only, by column). (E) Line profiles (white, dotted line in (D)) for each expected material. Note the PCD only line profile is taken from (C).

Fig 12 summarizes the results of material decomposition, which is performed following regularized, iterative reconstruction (Fig 1, step 19; Fig 7, step 14). Specifically, the resultant material maps are overlaid and coded by color (material) and intensity (concentration, fraction of water). Each column corresponds with the central 2D slice through a different material disk. Comparing the expected material maps (A) with the PCD only material maps produced following algebraic initialization (B) (produced for reference only), there is a clear need for regularization to separate the component materials. Comparing (B) with (C), the fidelity of the material decomposition results improves dramatically following regularized, iterative reconstruction. Further improvement is seen in the final hybrid reconstruction results (D), with an appreciable reduction in background contamination from the barium map (green) and improved visibility of the spherical lesions.

Insets specific to a single material map (yellow boxes in column 1; single 2D slices) compare the set of line pairs with a frequency of 2.84 lp/mm between the expected decomposition results (top) and the final hybrid decomposition results (bottom). Given that this set of line pairs is not resolved in the PCD only data (C), a fusion of the EID spatial resolution and the PCD spectral contrast is required to resolve this feature in the material decomposition. Visually, the calcium line pairs are well resolved; however, the iodine and barium line pairs are somewhat degraded relative to the expected results. Specifically, the iodine line pairs appear to represent the correct spatial frequency but, on close inspection, reverse the expected positive and zero lines (i.e. the enhanced lines are out of phase). The barium bars appear to represent the correct phase, but the bars are not uniformly resolved. Notably this inconsistency by material disk likely explains the variability in the aggregated MTF measurements shown in Fig 11F.

The final part of Fig 12(E) shows line profiles drawn through the expected material maps (dashed white line in (D) through 2.0 mm lesions). The line profiles are compared between the expected (A), PCD only (C), and hybrid decomposition results (D). The calcium line profiles closely match the expected line profiles in the PCD only reconstruction across all concentrations, with a modest improvement in the hybrid reconstruction results. Notably, the PCD only and hybrid decomposition errors appear correlated, which is expected given that the same PCD projection data was used for both reconstructions. For iodine, the noise level in the hybrid line profile is significantly reduced relative to the PCD only line profile; however, the concentration is clearly underestimated at 15 mg/ml and 3 mg/ml. The barium line profile is the most degraded relative to the expected results; however, the PCD only line profile is appreciably noisier than the hybrid line profile. In both profiles, the reliability of the barium measurements appears to fall off significantly below 10 mg/ml. This is likely related to cross-contamination with the iodine map (D) and to the greater noise level in the energy thresholds used to sample the barium K-edge contrast (37, 39 keV vs. 34 keV for iodine).

### *In vivo* experiment

**Fig 13:**
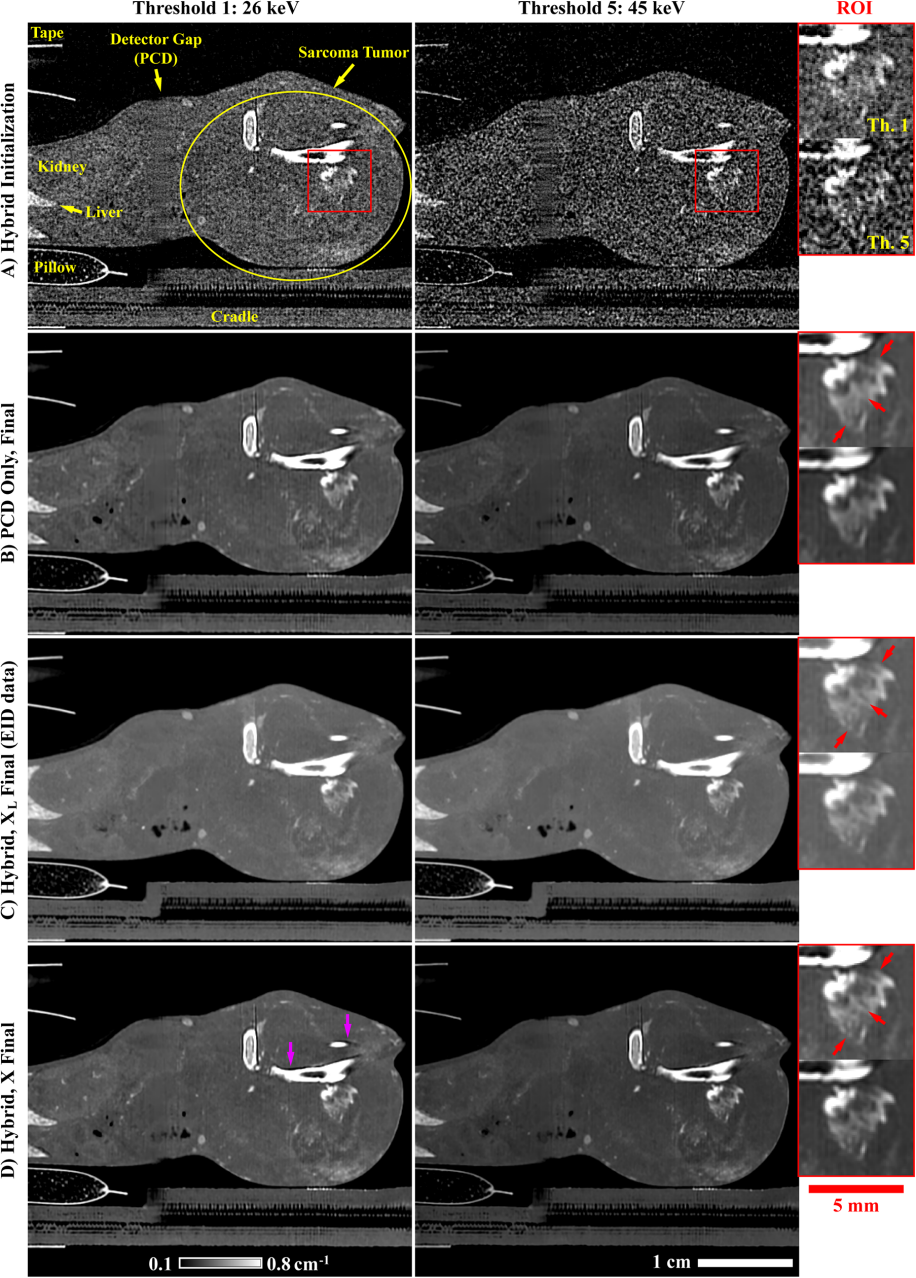
Hybrid, spectral CT reconstruction in an *in vivo* mouse model of soft tissue sarcoma. (A) 2D, sagittal slice through the algebraic initialization results shown for the least noisy data set (26 keV threshold) and the most noisy data set (45 keV threshold) by column (Fig 1, step 5; X_L_ + X_S_). A yellow oval denotes the location of the sarcoma tumor on the flank of the mouse. Red squares denote a region of interest (“ROI”) which is magnified, at right, for both thresholds. (B) Final PCD only reconstruction results (X; 6 iterations of regularized reconstruction). (C) Final hybrid reconstruction results for X_L_ (6 iterations of regularized reconstruction). Red arrows within the magnified region of interest denote high-contrast features which appear to be better resolved in the hybrid reconstruction. (D) Final hybrid reconstruction results for X = X_L_ + X_S_. Magenta arrows denote attenuation artifacts around bone.

Fig 13 summarizes the results of applying the hybrid spectral CT reconstruction algorithm to the EID and PCD projection data acquired in a mouse model of soft tissue sarcoma (*In vivo experiment* sub-section of the *Methods*). Concentrating on the energy thresholds with the lowest (26 keV) and highest (45 keV) noise levels (columns), Fig 13A displays algebraic initialization results in matching, 2D sagittal slices through the sarcoma tumor (located on the flank of the mouse, yellow oval). Notably, this comparison is analogous to Fig 8D, which compares algebraic initialization results in the digital phantom for the same thresholds. As in the simulation experiment, the need for regularization following component substitution and initialization is clear. Fig 13B shows matching slices through the final reconstruction results using the PCD data only. Finally, Fig 13C and 13D show matching slices through the final hybrid reconstruction results for the EID data (X_L_) and the PCD data (X = X_L_ + X_S_). In Fig 13D, magenta arrows point to attenuation artifacts visible around dense bone. Careful inspection reveals that these artifacts are visible in the initialization (A), the PCD only reconstruction (B), and in the hybrid reconstruction results (D), but are less obvious in the hybrid EID reconstruction (C). There are several possible explanations for this artifact. The most likely relate to imperfect modeling and inversion of the blurring operator and to log-transformation of energy-dependent line integrals. Red boxes in Fig 13A denote a region of interest (ROI) defined around a high contrast feature which is magnified at the right of each row. Comparing the PCD only results (B) with the hybrid results (C, D) within this ROI, red arrows denote apparent improvements in spatial resolution achieved through hybrid reconstruction. Notably, this magnified ROI also highlights the success of the proposed regularization strategy in enforcing redundancy between energies, despite differences in attenuation and significant initial differences in noise level (A).

**Fig 14:**
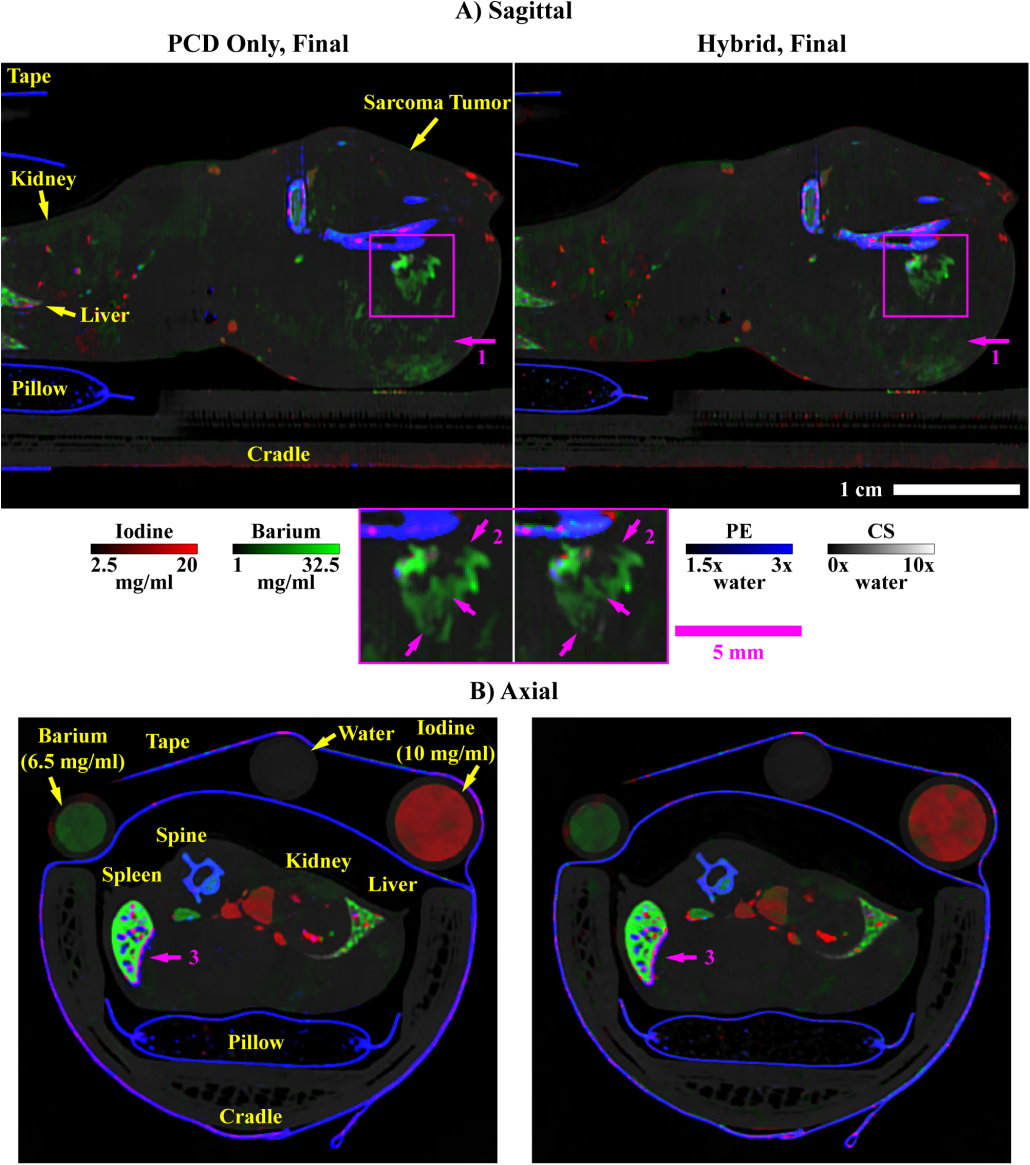
*In vivo* material decomposition. (A) PCD only (left) and hybrid (right) decomposition results for the 2D sagittal slice shown in each panel of Fig 13. Magenta arrows in the main panels (1) denote a region of barium enhancement within the tumor where the concentration appears lower in the hybrid results than in the PCD only results. Magenta boxes and the arrows within them (2) refer to the same ROI and features as in Fig 13. (B) Axial slice through the left kidney, liver, spleen, and spine of the mouse. Yellow labels and arrows denote three material calibration vials used to calibrate the material sensitivity matrix. A magenta arrow near the spleen (3) denotes material decomposition errors which result from physiological motion between scans.

Fig 14 continues the comparison between the final PCD only reconstruction results (left) and the final hybrid reconstruction results (right) following material decomposition. The sagittal slices and the ROI in Fig 13A match the sagittal slices and ROI analyzed in Fig 14. While the differences between the PCD only and hybrid decomposition results are subtle, careful inspection of the barium accumulation within the tumor reveals lower apparent concentrations in the hybrid barium map (magenta arrows, 1). Within the magenta ROI, the apparent resolution differences between the PCD only and hybrid data are seen to appear in a feature composed of extravasated barium nanoparticles within the sarcoma tumor. The visibility of the differences in this feature is significantly enhanced in the color-coded decomposition results (magenta arrows, 2). Fig 14B compares, axial 2D slices through the left kidney, liver, spleen, and spine of the mouse in the PCD only (left) and hybrid (right) decomposition results. This particular slice shows a cross-section through the barium, water, and iodine vials used to calibrate the material sensitivity matrix for the *in vivo* data (*In vivo experiment* sub-section of the *Methods*). It also shows material decomposition errors near the spleen resulting from physiological motion between scans (magenta arrows, 3). As expected for larger nanoparticles which are slowly cleared via the liver and spleen, significant barium accumulation is observed in these organs three days after injection [68]. Similar to the phantom simulation results in Fig 12, some degree of cross-contamination is observed between the barium and iodine maps within the calibration vials and in the vasculature within and around the kidney.

**Fig 15:**
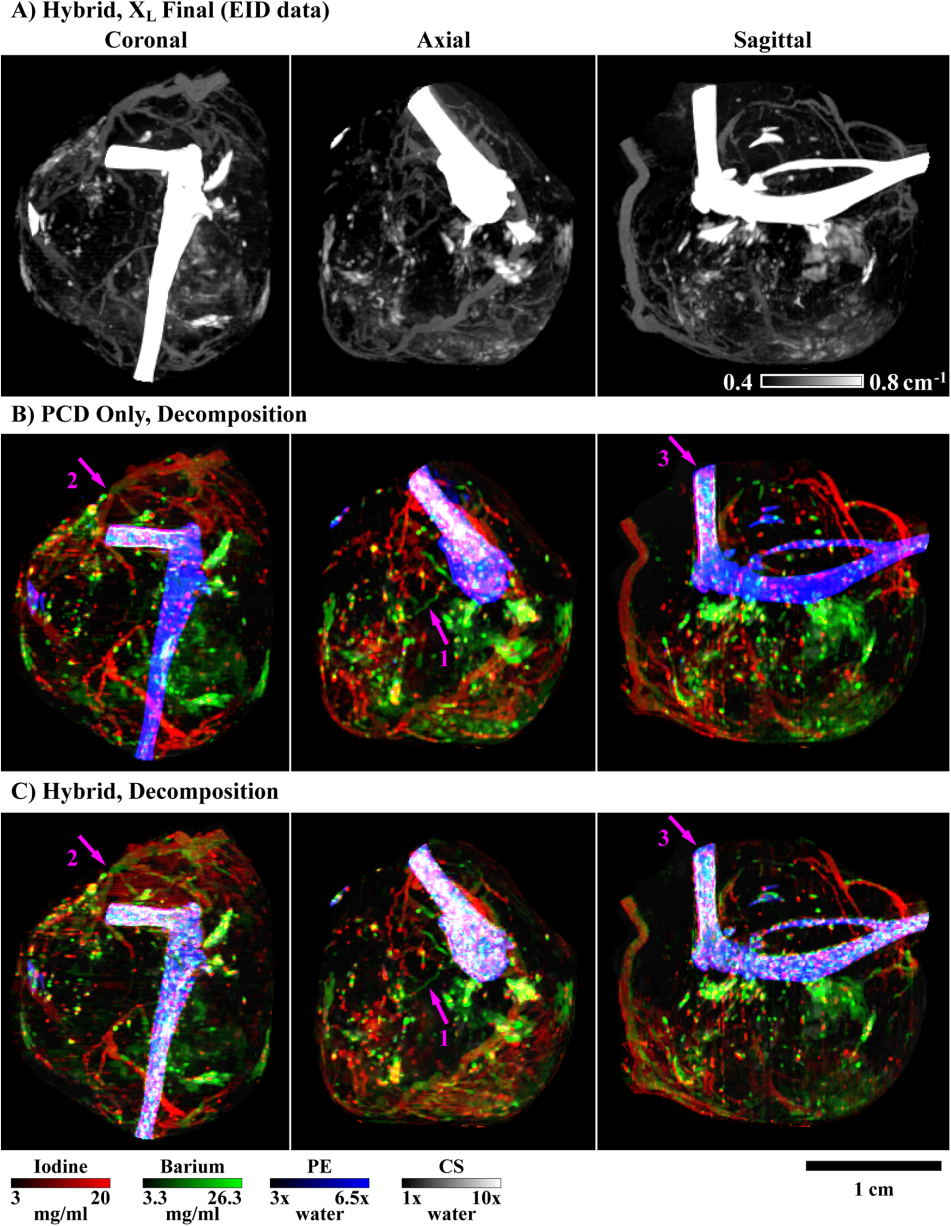
*In vivo* material decomposition (continued). (A) Maximum intensity projections (MIPs) computed through the segmented sarcoma tumor using the X_L_ component of the final hybrid reconstruction results. The same tumor is shown in coronal, axial, and sagittal orientations. (B) Comparable MIPs through the material decomposition of the final PCD only reconstruction results. (C) Comparable MIPs through the material decomposition of the final hybrid reconstruction results. Magenta arrows in (B) and (C) denote features of interest: (1) potentially misclassified vessels; (2) iodine artifact resulting from localized misregistration between the EID and PCD data; and (3) apparent material decomposition errors around bone.

Fig 15 continues the analysis of the *in vivo* material decomposition results in maximum intensity projections (MIPs) computed through the sarcoma tumor following manual segmentation. For reference, Fig 15A shows MIPs computed through the X_L_ component of the final hybrid reconstruction results in coronal, axial, and sagittal orientations. Panels (B) and (C) show analogous MIPs through material decomposition maps computed using the PCD only and hybrid reconstructions, respectively. Comparing the EID reconstruction with the material decompositions, the value of spectral CT in separating extravasated barium from vascular iodine and in separating bone from exogenous contrast is made clear. The tumor’s heterogeneity and local vascular permeability are highlighted by the accumulation of barium nanoparticles, approaching measured concentrations as high as 30 mg/ml in certain regions. Iodine is present in lower concentrations, ranging from 8–15 mg/ml, and highlights tumor vasculature with a range of diameters from ~0.3–1.2 mm. Notably, reliable (though possibly bias) detection of these concentrations of iodine and barium is expected based on the simulation results summarized in Fig 12. Accurate separation of iodine and barium in the smaller diameter vessels is questionable, however, based on the simulation results. Magenta arrows in Fig 15B and 15C (1) point to two small vessels which appear to be misclassified as containing barium rather than the expected iodine. Further sources of uncertainty are similarly highlighted by magenta arrows. Comparing the PCD only results with the hybrid results, an artifact is seen in the iodine map of the hybrid decomposition which is not present in the PCD only decomposition (2). The source of this artifact is localized misregistration due to motion between the sequential EID and PCD scans. Another set of arrows (3) points to apparent material decomposition errors around bone which are likely related to some real amount of vascularity within the bone and to the attenuation artifacts around the bone (Fig 13). Given these apparent errors, some amount of morphological dilation would be required to use the PE map to automate the separation of bone from contrast material.

### Computational considerations

As previously mentioned, the reconstruction code used for the simulation experiment was identical to the reconstruction code used for the *in vivo* experiment. The reconstruction code was executed from MATLAB (The MathWorks, Inc., Natick, MA) and called GPU-based CUDA kernels (NVIDIA Corporation, Santa Clara, CA; CUDA toolkit v6.5) for all distance-driven projection and backprojection operations [69] and for all resampling, BF, and OMP operations. The code was executed on a stand-alone workstation with a Core i7 processor clocked at 3.00 GHz (Intel Corporation, Santa Clara, CA), 64 GB of RAM, and a GeForce GTX Titan X graphics card with 12 GB of onboard memory (NVIDIA Corporation, Santa Clara, CA). For the *in vivo* results, the total computation times were 22.4 for the PCD only reconstruction and 36.2 hours for the hybrid reconstruction. Specifically, these computation times correspond with the time required to perform component substitution (hybrid only), initialization, and 6 iterations of regularized reconstruction with the split Bregman method. Negligible additional computation time was associated with material decomposition. For the PCD only reconstruction, 5 total volumes were reconstructed, corresponding with each PCD energy threshold (dimensions: 608x608x816 voxels). For the hybrid reconstruction, 5 additional volumes of the same dimensions were reconstructed from the EID projection data, one for each energy threshold (columns of X_L_).

Notably, regularization with BF and OMP accounted for less than 5% of the total computation time for both the PCD only and hybrid reconstructions. The bulk of the execution time was associated with the data fidelity update steps and execution of the BiCGSTAB solver (Fig 1, steps 5 and 18; Fig 7, steps 2 and 13). During initialization, the BiCGSTAB solver was run for 30 iterations per energy threshold. During regularized, iterative reconstruction, the BiCGSTAB solver was run for 25 iterations per energy threshold and per Bregman iteration. For reference, reconstruction of the digital phantom took 5.5 hours for the PCD only data and 8.9 hours for the hybrid data (volume dimensions: 440x440x308 voxels).

## Discussion

In this work we have proposed a hybrid spectral CT reconstruction technique designed to fuse the spatial resolution and signal-to-noise properties of EID data with the energy resolution and spectral contrast of PCD data. The objective is to benefit from the superior material discrimination provided by PCD hardware while overcoming some of the physical limitations on PCD pixel size and photon flux. Theoretically, the proposed algebraic reconstruction technique combines a novel, hybrid data fidelity constraint, which enforces agreement between the two sets of projection data, with a dual constraint on spectral rank and joint intensity gradient sparsity. This rank-sparsity constraint is enforced with a simplified version of our previously proposed regularization scheme, RSKR. Further regularization with respect to an empirically learned signal model for the high-resolution EID data is achieved through the application of dictionary learning and sparse coding. Practically, the proposed technique is made possible through several supporting algorithms, including those for high-fidelity geometric calibration between the EID and PCD imaging chains and for preprocessing the projection data. Equally important for this technique are continuing advancements in hardware and software for massively parallel computing.

### Stages of hybrid spectral CT reconstruction

A significant property of the proposed technique is that it can be executed in three distinct stages depending on the difficulty of the reconstruction problem, the allowable computation time, and the ultimate objective of hybrid reconstruction. As demonstrated in Fig 8, the first stage, component substitution, visually combines the high-resolution details of the EID data with the spectral contrast of the PCD data. The computational cost associated with this step is trivial, requiring only FBP reconstruction and least-squares material decomposition. Given a sufficiently well-conditioned material decomposition problem and low levels of noise in the reconstructed data, this strategy is a natural extension of analytical reconstruction to the problem of hybrid spectral CT. While potentially suitable for contrast enhancement and for overcoming field-of-view limitations, this approach will not reliably improve resolution in material decomposition results, since no deblurring is performed, and it is prone to noise amplification based on the conditioning of the material decomposition problem.

The second stage refines the results of component substitution with unregularized iterative reconstruction to reconcile the EID and PCD projection data in the image domain. Given a model for the spatial resolution differences between the EID and PCD data, some combination of deblurring and post-reconstruction regularization would be performed to address noise and to improve spatial resolution in the results of material decomposition. In our proposed algorithm, preliminary deblurring is performed during algebraic initialization, while “post-reconstruction” regularization is performed during the first iteration of regularized reconstruction. This second stage may strike an appropriate balance between computation time and diagnostic value in future clinical applications.

The third stage, which we have demonstrated in this work, uses regularized, iterative reconstruction to perform deblurring and denoising, managing spectral bias through data fidelity updates. While computationally expensive for realistic CT reconstruction problems, this third stage may be necessary for working with PCD projection data with highly variable levels of noise between thresholds. It may also be necessary to achieve satisfactory material decomposition results when attempting to separate two or more K-edge contrast agents near the energy resolution limits of the detector hardware (e.g. iodine and barium). *In vivo* micro-CT data in preclinical imaging applications, which is often an order of magnitude noisier than clinical CT data, will also greatly benefit from regularized, iterative reconstruction.

### Regularization of spectral CT data

Spectral CT imaging applications stand to benefit greatly from an emerging class of regularization schemes which synergize the power of sparse representation with the power of structural redundancy. Experimenting with this synergy to perform the largely unprecedented separation iodine and barium at *in vivo* concentrations, we employed two regularization schemes: RSKR and dictionary learning with sparse coding. Each of these regularization strategies has unique properties which are highly attractive for spectral regularization. RSKR is an extension of joint BF, which enforces matching intensity gradient sparsity patterns between spectral data sets with robustness to contrast differences and variable noise levels between data sets. Exploiting the expectation of a low-rank material basis for spectral contrast, RSKR iteratively applies joint BF to weighted singular vectors, prioritizing the preservation of high fidelity data while regressing poorly resolved data to be structurally dependent. Though powerful, RSKR has two notable weaknesses: (1) the domain size of the filter limits the spatial frequencies in which noise is addressed, and (2) the piece-wise constant signal model implied by intensity-gradient sparsity does not strictly model smooth edges in band-limited CT data. In this work, we attempted to address the domain size limitation (1) through a simple and effective multi-resolution strategy we call tiling. To overcome the inexact match between the results of RSKR and the intrinsic signal model of the data (2), we performed sparse coding with OMP. RKSR served as a preprocessing step for sparse coding, improving redundancy at the level of whole volumes prior to fitting the data with 3D structural patches from the pre-trained dictionary. For the problem of hybrid spectral CT reconstruction, we further exploited the power of dictionary encoding to reproduce an empirically learned signal model by enforcing the expected EID signal model on the reconstructions of the PCD data.

Based on the success of this sequential application of RSKR and OMP, we believe the next advancements in spectral regularization will be derived from a formalized, multi-resolution approach to jointly enforcing sparse representation and structural redundancy. The multi-resolution aspect of this approach will be key to simultaneously exploiting redundancy at the level of whole volumes, where there is a high degree of statistical power in grouping linearly dependent voxels, and at the level of localized patches, where the rank of the data will tend to be much lower than the number of spectral samples. Drawing from existing literature, we believe this advancement will combine the strengths of multi-resolution spatial transformation in sparse representation (e.g. wavelet transforms, tight-frame transforms [27]), of empirical learning of the relationships between spatial and spectral intensity variation and artifacts (e.g. tensor dictionaries [50], discriminative feature representation [70, 71]), and of computationally and memory efficient methods for processing volumetric image data (e.g. neural networks [72]).

### Parameter selection

A strength of the proposed hybrid spectral CT reconstruction technique is that it provides analytical formulas for deriving many of its parameters (e.g. regularization parameters in Eqs 22 and 23; regularization strength scaling in Eq 51). These scaling formulas should allow the technique to achieve good performance in a range of hybrid reconstruction problems with minimal effort spent on parameter tuning. That said, the performance of the technique can still be fine-tuned for specific applications through user-specified parameters which govern trade-offs between denoising performance, spatial resolution preservation and enhancement, and material decomposition fidelity. Table 1 summarizes these parameters along with the subsections of the paper in which they are introduced or otherwise defined. Notably, the parameter values in Table 1 were used for both the simulation and *in vivo* experiments presented in this work.

**Table 1:** User-specified parameters.

Manuscript Subsection	Parameter	Description	Value Used
**Hybrid data fidelity**			
	η	weighted least-squares (EID & PCD)	3
	g_*i*_	for all *i* (EID & PCD)	1
**Penalized algebraic reconstruction**			
	λ_C,*e*_	hybrid data fidelity; for all *e*	1
	α	regularization scaling factor	0.01
**Joint bilateral filtration**			
	b	BF domain radius	6
**RSKR**			
	h_0_	noise multiplier	1.5
	γ	regularization scaling exponent	0.5
	s	voxel stride used for tiling	3
**Dictionary learning**			
	b	3D patch radius	4
	s	voxel stride for noise measurement	3
	n_a_	dictionary atom count	1024
K-SVD algorithm			
	n_0_	max non-zero coefficients	48
		number of patches used	500,000
		K-SVD iterations	25
regularization with OMP			
	n_0_	max non-zero coefficients	5
**Computational considerations**			
initialization (Fig 1, step 5)		BiCGSTAB iterations, per energy	30
each Bregman iteration (Fig 1, step 18)		BiCGSTAB iterations, per energy	25
(Fig 1, steps 13–18)		total number of Bregman iterations	6

To guide future optimizations, adaptations, and modifications of our technique, several parameters in Table 1 deserve special attention (in addition to the points already raised in their respective subsections). The weighted least-squares parameters listed under *Hybrid data fidelity*, η and g_*i*_, (Eq 4) are nominally calibrated based on repeated measurements taken with specific detector hardware [39]. Practically, however, an undesirable side-effect of such calibration appears to be some loss of spatial resolution around highly attenuating features. Therefore, instead of using detector calibrated hardware parameters (i.e. η ≈ 0.5 in this work), we used a much more conservative parameter for both the EID and PCD data (η = 3). As in our previous work [36], when this more conservative value is used, the results of algebraic initialization are largely unchanged relative to unweighted least-squares. What does change is the effective regularization strength as a function of attenuation. In other words, least-squares weighting indirectly adjusts the scalar regularization parameters (μ_L,*e*_, μ_S,*e*_; Fig 1, step 18) on a per-line-integral basis.

As previously discussed, the *RSKR* parameters listed in Table 1 control the denoising strength (h_0_), the relative contributions of the EID and PCD data to the jointly constructed range kernel (γ), and the balance between the potential for spectral bias and the suppression of correlated noise (s). The values for these parameters were chosen empirically, based on past work [14, 36, 38], and quantitatively through the type of RSME analysis presented in Figs 10 and 12. These parameter values should serve as a good starting point for most spectral denoising applications; however, future work should focus on better optimizing these parameters for spatial resolution transfer between EID and PCD data and between the EID data and the resultant material decompositions. In contrast to RSKR, which was developed specifically for regularization of multi-dimensional CT data, the *Dictionary learning* methods and parameters employed in this work were largely based on a growing body of literature demonstrating the value of learned dictionaries in denoising and signal enhancement applications. We believe that some of the specifics of our approach such as regularization strength scaling based on the algorithmically measured noise level (Eq 54) and the application of a dictionary learned on the EID to denoising lower-resolution PCD data represent valuable contributions; however, future work should focus on adapting more recent concepts from dictionary learning literature (e.g. tensor dictionaries [50], discriminative feature representation [70, 71]) to the problem of resolution enhancement in hybrid spectral CT reconstruction.

### Limitations and future work

In this work, we have made several assumptions and simplifications to derive our hybrid spectral CT reconstruction technique and to apply it to *in vivo* data. With respect the EID and PCD data, we have assumed that a single set of EID projections is acquired at a higher spatial resolution and a lower level of noise than each threshold of the PCD data. While we have demonstrated that this paradigm can improve material decomposition results (Fig 12), practical radiation dose constraints will require a compromise between the fidelity of the EID and PCD data for *in vivo* applications. A prime example of this consideration in practice is the field-of-view extension strategy employed with the Mayo clinic prototype scanner [9]. Future work in the area of hybrid CT reconstruction should consider appropriate dose allocation between the EID and PCD imaging chains for applications such as field-of-view extension to overcome limitations in PCD detector size, signal-to-noise ratio enhancement for improved material decomposition, and spatial resolution enhancement.

To make iterative, hybrid reconstruction computationally tractable in large 3D data sets, we adopted simple models for the energy dependence of x-ray attenuation and for spatial resolution in cone-beam CT reconstructions. For the spectral model, we employed log-transformation to linearize the CT reconstruction problem. For EID data acquisition and reconstruction, the consequences of representing attenuation coefficients using the monoenergetic form of Beer’s law are well documented and often accepted in practice [73]. For PCD data, however, where the response of the detector is known to vary significantly with energy [16], this assumption is questionable, particularly when the ultimate goal is to perform quantitative material decomposition. Component substitution and hybrid iterative reconstruction will undoubtedly benefit from extensions that incorporate more precise, energy-dependent forward models of the imaging process. A similar simplification was the treatment of the point spread function as a spatially-invariant Gaussian in both the EID and PCD data. While this may be a reasonable approximation for the in-plane spatial resolution of the EID data, it undoubtedly limited deblurring performance for the PCD data which was acquired at higher geometric magnification. Notably, we did minimize the impact of the loss of resolution on our *in vivo* results by aligning the sarcoma tumor with the central ray of the x-ray source and detector and with the center of rotation for both imaging chains.

Advancements in the implementation of hybrid reconstruction and in parallel computing will make the incorporation of more complex and accurate energy and spatial resolution models feasible. For now, however, the computation time and memory allocation associated our proposed hybrid algorithm is significant (*Computational considerations* sub-section). To make the current implementation more tractable in terms of system memory requirements, we made an algorithmic simplification which deviates from the formalism of the split Bregman method. Specifically, rather than track independent sets of regularization variables (D) and residuals (V) for each form of regularization (RSKR, OMP), we only tracked one set after applying both forms of regularization. With respect to the computation time, more than 95% of the total time was associated with the algebraic initialization and the data fidelity update steps. Therefore, in future work hybrid reconstruction can be significantly accelerated using state-of-the-art iterative solvers [74] and more efficient implementations of the distance driven projection and backprojection operators [75]. Given that the data fidelity update steps are evaluated for each energy independently within the split Bregman framework, further computational speed-ups will be derived from multi-GPU parallelization.

On a final note regarding the limitations of our *in vivo* experiment, we point out two subtle, but potentially significant, details. First, while one goal of this paper is to present a regularization scheme applicable to hardware-binned PCD data, the PCD detector we simulated and used in our *in vivo* experiment has a single hardware threshold (PILATUS3 CdTe 300K; Dectris AG, Baden-Dättwil, Switzerland). We collected and used PCD data acquired at several different thresholds, without the subtraction between thresholds which is typically performed to yield binned data. This means that there was no explicit correlation in the number of counts per bin (per threshold) in our data as there would be for a single scan acquired with multiple hardware-based energy thresholds. Second, our primary motivation for pursuing hybrid spectral CT reconstruction is to overcome limitations in photon counting hardware through joint reconstruction with energy integrated data. In this work we focused on a realistic material decomposition problem with modest differences in spatial resolution and detector pixel sizes between the PCD and EID data (PCD: 172 μm^2^ pixels; EID: 88 μm^2^ pixels). Future work should characterize the performance of hybrid reconstruction over larger resolution and pixel size differences, with a particular focus on the adaptation of clinical PCD hardware (i.e. pixel size ~0.5 mm^2^) to preclinical imaging applications through joint reconstruction with high-resolution, EID projection data [14]. Establishing a link between PCD hardware developed with the financial incentive of clinical applications and existing EID-based preclinical micro-CT will promote translation between the two domains while advancing the state of the art in preclinical imaging.

## Conclusions

In this paper, we have proposed a novel technique for the joint reconstruction of projection data acquired with EIDs and PCDs, nominally overcoming differences in spatial resolution and noise through forward modeling and rank-sparse regularization. We believe that this and related techniques will be invaluable to overcoming several of the physical limitations on current PCD technology, facilitating validation and widespread adoption. Additionally, we believe that hybrid reconstruction techniques will be crucial for translating advancements in PCD data acquisition and processing between clinical and preclinical applications.
